# Kinectin1 depletion promotes EGFR degradation via the ubiquitin-proteosome system in cutaneous squamous cell carcinoma

**DOI:** 10.1038/s41419-021-04276-5

**Published:** 2021-10-23

**Authors:** Ji Ma, Shudong Ma, Ying Zhang, Yi Shen, Lei Huang, Tianhao Lu, Lu Wang, Yunhan Wen, Zhenhua Ding

**Affiliations:** 1 Department of Radiation Medicine, Guangdong Provincial Key Laboratry of Tropical Disease Research, School of Public Health, Southern Medical University, 510515 Guangzhou, China; 2 Department of Oncology, Nanfang Hospital, Southern Mediacal University, 510515 Guangzhou, China; 3 Department of Burn, Nanfang Hospital, Southern Mediacal University, 510515 Guangzhou, China

**Keywords:** Squamous cell carcinoma, Proteasome

## Abstract

Depletion of kinectin1 (KTN1) provides a potential strategy for inhibiting tumorigenesis of cutaneous squamous cell carcinoma (cSCC) via reduction of epidermal growth factor receptor (EGFR) protein levels. Yet, the underlying mechanisms of KTN1 remain obscure. In this study, we demonstrate that KTN1 knockdown induces EGFR degradation in cSCC cells by promoting the ubiquitin-proteasome system, and that this effect is tumor cell-specific. KTN1 knockdown increases the expression of CCDC40, PSMA1, and ADRM1 to mediate tumor suppressor functions in vivo and in vitro. Mechanistically, c-Myc directly binds to the promoter region of CCDC40 to trigger the CCDC40-ADRM1-UCH37 axis and promote EGFR deubiquitination. Furthermore, KTN1 depletion accelerates EGFR degradation by strengthening the competitive interaction between PSMA1 and ADRM1 to inhibit KTN1/ADRM1 interaction at residues Met1-Ala252. These results are supported by studies in mouse xenografts and human patient samples. Collectively, our findings provide novel mechanistic insight into KTN1 regulation of EGFR degradation in cSCC.

## Introduction

The ubiquitin-proteasome system (UPS) consists of two coupled pathways: ubiquitination and proteolysis. Hydrolysis of proteins via the UPS is required for cellular homeostasis [1]. The UPS acts as a nonspecific recycling route to degrade over 80% of cellular proteins, mostly including short-lived and regulatory proteins or damaged and misfolded proteins that enter into the proteasomal passage [2]. Proteins designated to be proteolyzed are covalently conjugated with ubiquitin (Ub) at the α amino group of lysine residues as a degradation tag. Proteasome signaling is activated after the poly-Ub chain is extended to at least four Ubs. The proteasome is a large protein complex with a molecular mass of >2.5 MDa that is located in the cytoplasm. The proteasome holoenzyme consists of a barrel-shaped 20S core particle (CP) with proteasome activity and two 19S regulatory particles (RPs) capping the ends of the CP. The RPs functionally control the translocation of ubiquitinated substrates [3–5]. The subunits of the CP are arranged as two outer α-rings and two inner β-rings. Each type of ring consists of seven corresponding α/β subunits (PSMA1–7, PSMB1–7). β1 (PSMB1), β2 (PSMB2), and β5 (PSMB5) subunits hold three distinct proteolytic activities consisting of caspase-like activity, trypsin-like activity, and chymotrypsin-like activity. The subunits of the RPs form a base with ATPase activity and a lid without ATPase activity. In the base, Rpt1 (PSMC2) and Rpt6 (PSMC5) participate in substrate binding, unfolding and translocation, whereas Rpt2 (PSMC1), Rpt3 (PSMC4) and Rpt5 (PSMC3) control the 20S gate. In the lid, Rpn1 (PSMD2), Rpn10 (PSMD4) and Rpn13 (ADRM1) serve as ubiquitin receptors and are necessary for binding of ubiquitylated substrates. The deubiquitylases (DUBs) USP14 and UCH-L5 (UCH37) deubiquitinate the substrates to activate the RP, and the DUB Rpn11 (PSMD14) removes the final Ubs [6]. Thus, the UPS is comprised of an intricate machinery that has many layers of regulation to accurately degrade proteins to maintain cellular homeostasis.

The epidermal growth factor receptor (EGFR, ERBB-1) is an oncogenic receptor protein tyrosine kinase (RPTKs). It is one of four HER (ERBB) family members located on the surface of the cytomembrane [7, 8]. EGFR is a glycoprotein that is activated by binding peptide ligands, such as epidermal growth factor (EGF) and transforming growth factor α (TGFα) [9]. EGFR activation triggers signaling that leads to its homodimerization or heterodimerization with HER2 and transphosphorylation of other ERBB members. In this process, another ERBB member, HER3, serves as a stabilized phosphotyrosine scaffold [10]. A series of biochemical processes orchestrated by EGFR activation leads to the stimulation of downstream pathways, including the RAS/RAF/MAPK, PI3K/Akt/mTOR and STAT transcriptional signaling pathways [11]. *EGFR* gene mutation is associated with aberrant EGFR protein expression in a wide range of human epithelial cancers. Evidence suggests that EGFR overexpression leads to tumor cell survival, proliferation, invasion and metastasis [12, 13]. Recent research also has demonstrated additional factors that participate in the EGFR pathway. T-cadherin (T-cad) serves as negative regulator of the EGFR pathway by controlling functional events of the EGFR activation cascade [14]. Moreover, GEP100 directly binds to Tyr1068/1086-phosphorylated EGFR through its pleckstrin homology domain to activate Arf6 [15]. EGFR expression may act as an indicator of disease recurrence or shorter patient survival in some cancers [16], especially in cutaneous squamous cell carcinoma (cSCC). Furthermore, the expression of EGFR has been reported to be upregulated in cSCC and to play a prominent role in cSCC proliferation, invasion, and metastasis [17, 18]. Thus, regulation of EGFR protein levels represents an effective potential strategy for anti-cancer therapy.

EGFR levels are known to be regulated in part by the UPS-mediated degradation pathway. The E3 ligase UBE4B promotes EGFR degradation by coupling the sorting machinery with ubiquitination [19]. In addition, the phosphatase and tensin homolog (PTEN) inactivation may cause destabilization of newly formed ubiquitin ligase Cbl complexes and therefore decrease EGFR ubiquitination [20]. Furthermore, DGKδ deficiency induces degradation of EGFR by modulating the expression of USP8 and accelerating its ligand, which is regulated by PKCα and Akt phosphorylation [21]. These studies strengthen the importance of the UPS in degradation of EGFR as a mechanism for regulating EGFR levels.

In our previous study, we demonstrated that EGFR protein expression levels are downregulated following Kinectin1 (KTN1) knockdown, whereas knockdown of KTN1 had no effect on the RNA expression of EGFR in cSCC cell lines. The results indicate that knockdown of KTN1 regulates EGFR expression at the posttranslational level [18]. KTN1 is an isoform of kinectin, a membrane anchor of the microtubule motor protein kinesin, and is primarily localized to the endoplasmic reticulum (ER) [22–24]. Despite limited reports on its functional mechanisms, multiple variants and a differential splicing pattern of KTN1 have been identified in human hepatocellular carcinoma [25]. A potential linkage has also been suggested between KTN1 and cancer, and KTN1has been proposed to participate in protein synthesis. Hanry Yu’s lab first demonstrated that KTN1 interacts with elongation factor-1δ (EF-1δ) to anchor the EF-1δ complex in the ER, while it also increases the synthesis of membrane or secretory proteins [26]. Additionally, Cinzia Progida’s lab demonstrated that Rab18 directly interacts with KTN1 to anchor the ER via kinesin-KTN1 for focal adhesion (FA) growth and dynamics [27]. KTN1 has been shown to directly bind kinesin, and a current research focus involves identifying inhibitors of kinesin spindle protein (KSP) as targets for multi-cancer therapy [28]. Furthermore, EGFR has also been shown to regulate KSP and its related molecules [29], suggesting that there may be some potential overlap in the KTN1 and EGFR pathways. However, whether KTN1 reduction regulates EGFR protein levels via synthesis or degradation remains unclear.

Here, we demonstrate that KTN1 depletion induces the degradation of EGFR protein in cSCC. We also demonstrate that KTN1 interacts with the CP base subunit PSMA1 and the CP lid subunit ADRM1; and that PSMA1 and ADRM1 interact with each other. Furthermore, the interaction between PSMA1 and ADRM1 is increased by KTN1 depletion, which suggests that the KTN1 binds competitively to ADRM1. KTN1 depletion activates the CCDC40-ADRM1-UCH37 axis to decrease the ubiquitinylation of EGFR, which is consistent with expression changes in mouse xenografts and human cSCC patient samples. Thus, our data support a model in which changes in the UPS caused by KTN1 depletion lead to EGFR degradation in vitro and in vivo. Our study expands the understanding of KTN1 function in protein degradation, which defines a new network of UPS in EGFR degradation in cSCC.

## Results

### KTN1 knockdown reduces EGFR protein expression post-transcriptionally and increases apoptosis to repress the cSCC cell survival

**Fig. 1:**
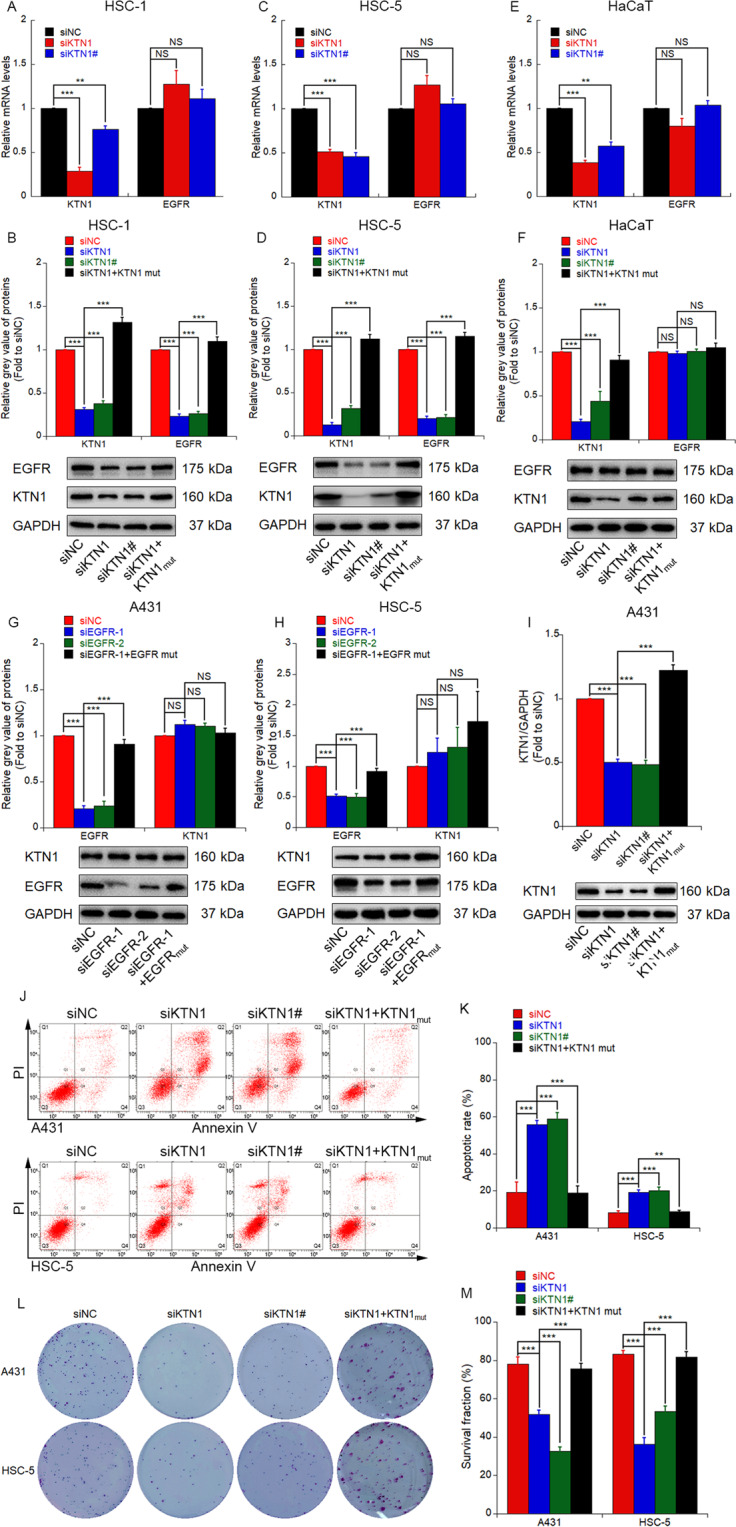
Knockdown of *KTN1* mediates post-transcriptional reduction of EGFR protein expression, increases apoptosis, and represses survival of CSCC cells. **A** HSC-1 cells were transfected with siNC, siKTN1 or siKTN1#, and total RNA were extracted and analyzed by qRT-PCR. qRT-PCR results represent the means ± SD. Representative of results from three biological replicates. **B** HSC-1 cells were transfected with siNC, siKTN1, siKTN1#, or co-transfected with siKTN1 and KTN1_mut_, total proteins were extracted and analyzed by immunoblotting. Quantified results represent the means ± SD. Representative of results from three biological replicates. **C** HSC-5 cells were transfected with siNC, siKTN1, or siKTN1#, and total RNA were extracted and analyzed by qRT-PCR. qRT-PCR results represent the means ± SD. Representative of results from three biological replicates. **D** HSC-5 cells were transfected with siNC, siKTN1, siKTN1#, or co-transfected with siKTN1 and KTN1_mut_, total proteins were extracted and analyzed by immunoblotting. Quantified results represent the means ± SD. Representative of results from three biological replicates. **E** HaCaT cells were transfected with siNC, siKTN1, or siKTN1#, and total RNA were extracted and analyzed by qRT-PCR. qRT-PCR results represent the means ± SD. Representative of results from three biological replicates. **F** HaCaT cells were transfected with siNC, siKTN1, siKTN1#, and co-transfected with siKTN1 and KTN1_mut_, total proteins were extracted and analyzed by immunoblotting. Quantified results represent the means ± SD. Representative of results from three biological replicates. **G**, **H** A431 and HSC-5 cells were transfected with siNC, siEGFR-1, siEGFR-2, or co-transfected with siEGFR-1 and EGFR_mut_, and total protein was extracted and analyzed by immunoblotting. Quantified results represent the means ± SD. Representative of results from three biological replicates. **I** A431 cells were transfected with siNC, siKTN1, siKTN1#, and co-transfected with siKTN1 and KTN1_mut_, total proteins were extracted and analyzed by immunoblotting. Quantified results represent the means ± SD. Representative of results from three biological replicates. **J** Flow cytometry analysis of apoptotic cells in A431 and HSC-5 cells transfected with siNC, siKTN1, siKTN1#, or co-transfected with siKTN1 and KTN1_mut_. **K** Apoptotic cell ratios of A431 and HSC-5 cells transfected with siNC, siKTN1, siKTN1#, or co-transfected with siKTN1 and KTN1_mut_. Apoptotic cell ratios represent the means ± SD. Representative of results from three biological replicates. **L**, **M** Colony formation assay and survival fractions of A431 and HSC-5 cells transfected with siNC, siKTN1, siKTN1#, or co-transfected with siKTN1 and KTN1_mut_. Survival fractions represent the means ± SD. Representative of results from three biological replicates.

In our previous study, we demonstrated that KTN1 is highly expressed in cSCC, where it serves as a modulator of EGFR protein levels [18]. To further investigate the function of KTN1, we performed a loss-of-function assay. KTN1 RNA interference oligos siKTN1and siKTN1, which target different regions of the wt KTN1 coding sequence, were used to knockdown KTN1 expression in HSC-1 and HSC-5 cSCC cell lines, and a control siRNA (siNC) was assayed in parallel, meanwhile, siKTN1 rescue experiments were carried out by reintroducing target mutated KTN1 (KTN1_mut_). Significant loss of KTN1 mRNA and protein levels in siKTN1-transfected cells was confirmed by qRT-PCR and Western blotting assays. Furthermore, in accordance with our previous study, EGFR protein levels were downregulated by KTN1 depletion, though there was no effect on the EGFR mRNA expression (Fig. 1A–D). These results suggest that knockdown of KTN1 inhibits EGFR post-transcriptionally.

To explore whether KTN1 may specifically regulate EGFR protein levels in tumor cell lines, we transfected siKTN1 and siKTN1# into the human immortalized keratinocytes cell line HaCaT, in addition, KTN1mut was also transfected into the cell line to demonstrate the gene-specific of siKTN1. Knockdown of KTN1 had no effect on EGFR mRNA or protein levels in HaCaT cells (Fig. 1E, F), which suggests that knockdown of KTN1 inhibits EGFR the protein translation process in a cell-specific manner. To further clarify whether EGFR may reciprocally regulate KTN1 expression, we transfected 2 EGFR targeted siRNAs to knockdown of *EGFR* in A431 and HSC-5 cSCC cell lines meanwhile, SiEGFR-1 rescue experiments were carried out by reintroducing target mutated EGFR (EGFR_mut_). Knockdown of *EGFR* had no effect on KTN1 protein expression, which suggests that KTN1 is an upstream mediator of EGFR, but that EGFR does not reciprocally regulate KTN1 expression (Fig. 1G, H).

In our previous study, we demonstrated that knockdown of KTN1 promotes cell proliferation, invasion and migration of cSCC [18]. To further investigate the effects of KTN1 in cSCC cell lines, we transfected A431 and HSC-5 cells with KTN1 siRNAs to knockdown the KTN1 protein levels (Fig. 1I) and then detected the apoptotic rate and survival fraction by annexin-propidium iodide dual fluorescent staining and colony formation assay meanwhile, RNAi rescue experiments were carried out by KTN1_mut_ reintroducing. The results revealed a higher apoptotic rate and lower survival fraction in the KTN1 siRNA-transfected groups compared to the siNC group (Fig. 1J–M). Collectively, these results reveal that knockdown of KTN1 reduces EGFR protein expression post-transcriptionally and serves as a tumor suppressor strategy by increasing apoptosis and repressing the survival fraction in cSCC cells.

### KTN1 regulates EGFR degradation via the UPS

To clarify the underlying mechanism of EGFR protein reduction upon transfection of KTN1 siRNA, we transfected A431 cells with siRNA and then detected the expression of EGFR at 0, 2, 4, 6, 12, 18, and 24 h of post-transfection. EGFR protein levels decreased significantly at 12 and 18 h of post-transfection with siKTN1 compared to siNC (Fig. S1A, B). Next, we co-treated the cells with cycloheximide (CHX) to inhibit protein synthesis. The EGFR protein levels still decreased significantly at 12 and 18 h after transfection in the siKTN1 + CHX group as compared to the siNC + CHX group (Fig. S1C, D, G). These results rule out the possibility that knockdown of KTN1 reduces EGFR expression at the level of protein synthesis.

**Fig. 2:**
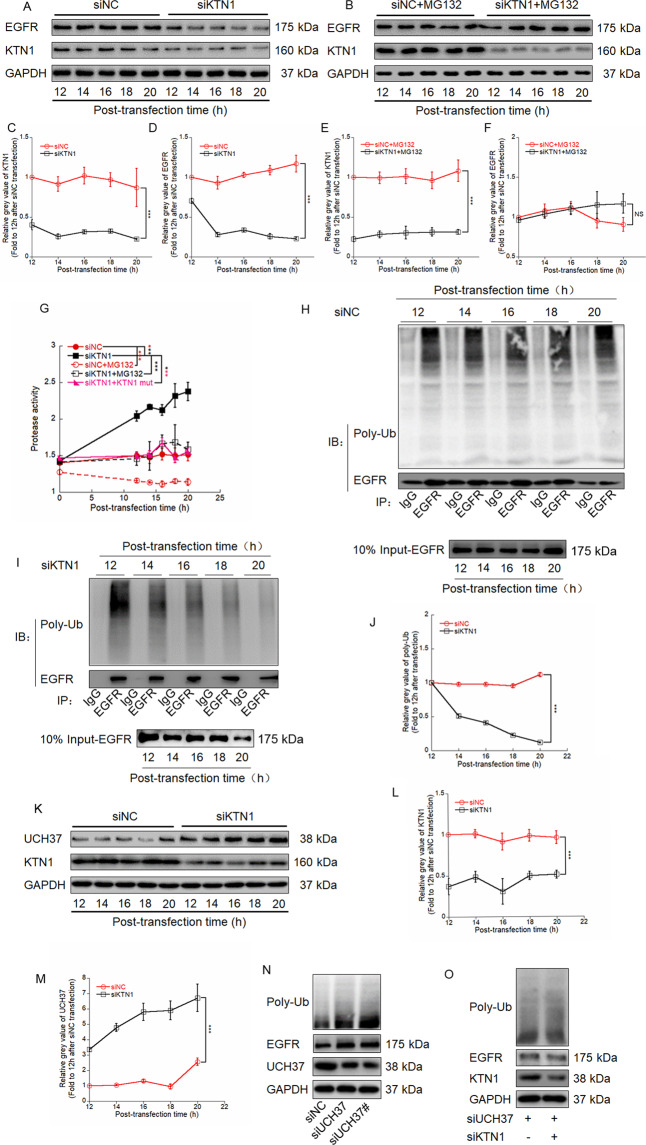
KTN1 regulates EGFR degradation via the UPS. **A** A431 cells were transfected with siNC or siKTN1 and collected after transfection for the indicated time periods. Total cell lysates were analyzed by immunoblotting with anti-KTN1 and anti-EGFR antibodies. Representative of results from three biological replicates. **B** Ten micromolar MG132 was added to siNC-transfected or siKTN1-transfected A431 cells, which were collected after transfection for the indicated time periods. Total cell lysates were analyzed by immunoblotting with anti-KTN1 and anti-EGFR antibodies. Representative of results from three biological replicates. **C**–**F** Gray value was analyzed by image J and to calculate Relative KTN1 or EGFR protein level. Quantified gray values represent the means ± SD. Representative of results from three biological replicates. **G** Protease activity assay of siNC-transfected cells, siKTN1-transfected cells or co-transfected with KTN1_mut_ without or with MG132 treated for the indicated time periods. The results of proteasome activity represent the means ± SD. Representative of results from three biological replicates. **H**–**J** A431 cells were transfected with siNC or siKTN1 and collected at the indicated times. Total cell lysates were analyzed by Co-IP with anti-EGFR antibody for IP and anti-poly-ubiquitin antibody for immunoblotting. Quantified gray values represent the means ± SD. Representative of results from three biological replicates. **K** A431 cells were transfected with siNC or siKTN1 and collected at the indicated times. Total cell lysates were analyzed by immunoblotting with anti-UCH37 antibody. Representative of results from three biological replicates. **L**, **M** Gray value was analyzed by image J and to calculate Relative KTN1 or UCH37 protein level. Quantified gray values represent the means ± SD. Representative of results from three biological replicates. **N** A431 cells were transfected with siNC, siUCH37, and siUCH37#. Total cell lysates were analyzed by immunoblotting with anti-UCH37, anti-EGFR, and anti-poly-ubiquitin antibodies. Representative of results from three biological replicates. **O** A431 cells were transfected with siKTN1 and/or siUCH37. Total cell lysates were subjected to immunoblotting with anti-KTN1, anti-KTN1, and anti-poly-ubiquitin antibodies. Representative of results from three biological replicates.

To further determine whether EGFR protein reduction after siKTN1 transfection is explained by effects on protein degradation, we applied MG132 after siRNA transfection to inhibit proteasomal activity. EGFR protein levels gradually decreased after transfection with siKTN1 compared to siNC in A431 cells; however, upon MG132 co-treatment, the effect of siKTN1 in decreasing EGFR protein levels was abrogated (Fig. 2A–F). Similar results were observed in HSC-5 cells (Fig. S2E, F, H–K). The ubiquitin-proteasome pathway (UPP) and autophagy-lysosome pathway (ALP) are main processes for protein degradation [30]. Therefore, we evaluated the effect of siKTN1 on the activity of proteases that mediate these pathways. Knockdown of KTN1 increased protease activity, which is consistent with its effect on the UPP (Fig. S2G).

Ubiquitination of target protein and polyubiquitin chain elongation are necessary for proteasome recognition and hydrolysis [31]. To directly determine whether EGFR is polyubiquitinylated in siKTN1-transfected cells, we performed Co-IP assays, which revealed that polyubiquitin co-immunoprecipitates with EGFR. We also found that the interacted polyubiquitin was decreased at 12–20 h after transfection of siKTN1 compared to siNC (Fig. 2H–G). Next, we detected the levels of UCH37, a deubiquitinating enzyme associated with the 19S regulatory subunit of the 26S proteasome. UCH37 expression gradually increased at 12–20 h after transfection of siKTN1 but not siNC, both in A431 cells (Fig. 2K–M) and HSC-5 cells (Fig. S1L–N). Further, to investigate whether knockdown UCHL5 may increase EGFR stability and ubiquitination, we transfected 2 UCH37 targeted siRNAs to knockdown of *UCH37* in A431, the results revealed that EGFR and polyubiquitin levels were upregulated by UCH37 depletion. Next, we co-treated the cells with siKTN1 to explore whether UCH37 is a key factor in knockdown KTN1 regulates EGFR stability. Knockdown of KTN1 and UCH37 had no effect on EGFR and polyubiquitin levels in A431 cells, which suggests that knockdown of UCH37 abrogated upregulated EGFR and polyubiquitin levels induced by knockdown of KTN1 (Fig. 2N, O). Collectively, these results demonstrate that knockdown of KTN1 promotes EGFR degradation via the UPS.

### *CCDC40*, *PSMA1*, and *ADRM1* are upregulated by KTN1 knockdown

**Fig. 3:**
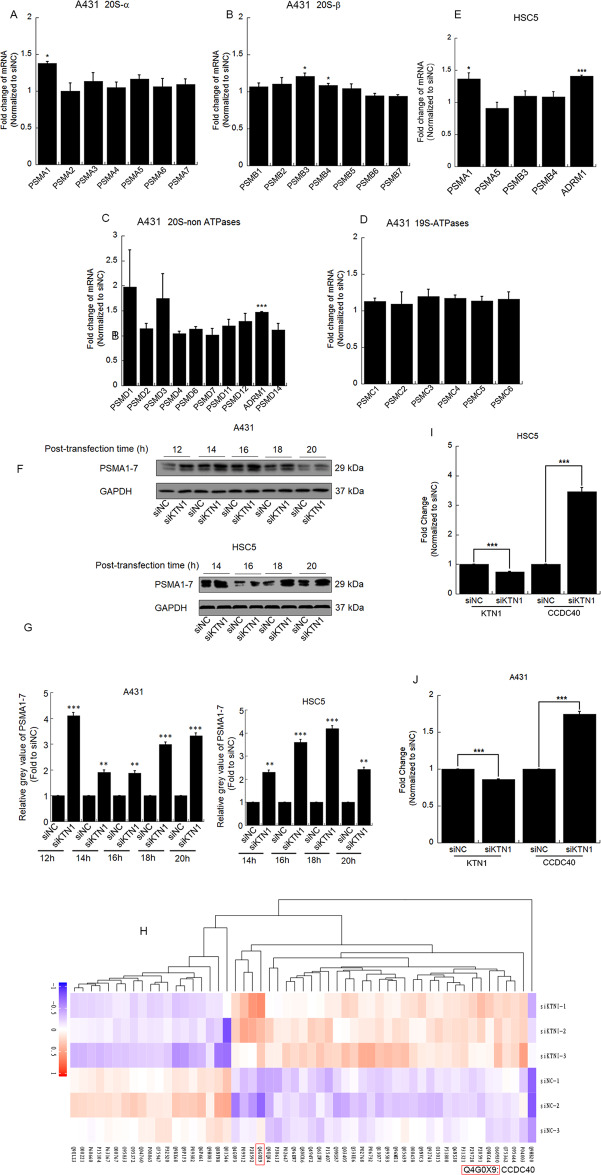
CCDC40, PSMA1, and ADRM1 are upregulated by knockdown of *KTN1*. **A**–**D** A431 cells were transfected with siKTN1. Total RNA was extracted, and mRNA of all proteasome subunits was detected by qRT-PCR. qRT-PCR results represent the means ± SD. Representative of results from three biological replicates. **E** HSC-5 cells were transfected with siKTN1. Total RNA was extracted, and PSMA1, PSMA5, PSMB3, PSMB4, and ADRM1 mRNAs were detected by qRT-PCR. qRT-PCR results represent the means ± SD. Representative of results from three biological replicates. **F** A431 or HSC-5 cells were transfected with siKTN1. Total cell lysates were collected at the indicated times and subjected to Western blotting. Representative of results from three biological replicates. **G** Gray value was analyzed by Image J. Representative of results from 3 biological replicates. **H** Differentially expressed proteins were clustered and shown in a heat map by hierarchical clustering analysis for proteomic profiles extracted from iTRAQ markers. The normalized log 2 of the fold change is represented in the heatmap by different colors. The maximum value is normalized to 1, and the minimum value is normalized to –1 to indicate the expression profiles of differential proteins under different experimental conditions. Representative of results from three biological replicates. **I**, **J** A431 or HSC-5 cells were transfected with siKTN1. Total RNAs were extracted, and CCDC40 mRNA was detected by qRT-PCR. qRT-PCR results represent the means ± SD. Representative of results from three biological replicates.

Next, we sought to explore the mechanism by which KTN1 knockdown may regulate EGFR degradation. Consequently, we performed qPCR to evaluate the levels of 20S and 19S proteasome subunits. In A431 cells, proteasome subunit α type 1 (PSMA1) of the 20S α-ring, proteasome subunit β types 3 and 4 of the 20S β-ring, and adhesion regulating molecule 1 (ADRM1) of the 19S ATPase were upregulated after transfection with siKTN1 versus siNC (Fig. 3A–D). Furthermore, upregulation of PSMA1 and ADRM1 after siKTN1 transfection was verified in HSC5 cells (Fig. 3E). The upregulation of PSMA1 could also be observed at the protein level by SDS-PAGE analysis followed by Western blotting (Fig. 3F, G). These results suggest that the EGFR degradation induced by knockdown of KTN1 may be mediated in part by increased proteasome activity.

To further explore the mechanism of EGFR degradation induced by knockdown of KTN1 in cSCC cell lines, we conducted isobaric tags for relative and absolute quantification (iTRAQ) in A431 cells transfected with siNC and siKTN1. Quality analysis demonstrated that three repeats provided strong consistency (Fig. S2A). Furthermore, base peak chromatography (BPC) suggested high quality of the iTRAQ samples (Fig. S2B). Analysis of differentially expressed proteins with *P*-value < 0.05 revealed 36 proteins that were upregulated, and 19 that were downregulated in siKTN1- versus siNC-transfected cells (Fig. 3H and Fig. S2C and Table S1), with the coiled-coil domain-containing protein 40 (CCDC40) representing the most highly upregulated protein after siKTN1 transfection. To verify these results and distinguish whether knockdown of KTN1 upregulates CCDC40 protein expression via transcriptional regulation or post-translation modification, we performed qPCR of CCDC40 mRNA levels. CCDC40 mRNA was upregulated after transfection with siKTN1 compared to siNC in both A431 and HSC-5 cells (Fig. 3I, J). Thus, these results indicate that knockdown of KTN1 promotes the transcriptional activation of CCDC40, PSMA1, and ADRM1.

### PSMA1 represents a key modulator of KTN1-mediated EGFR degradation in vivo and in vitro

**Fig. 4:**
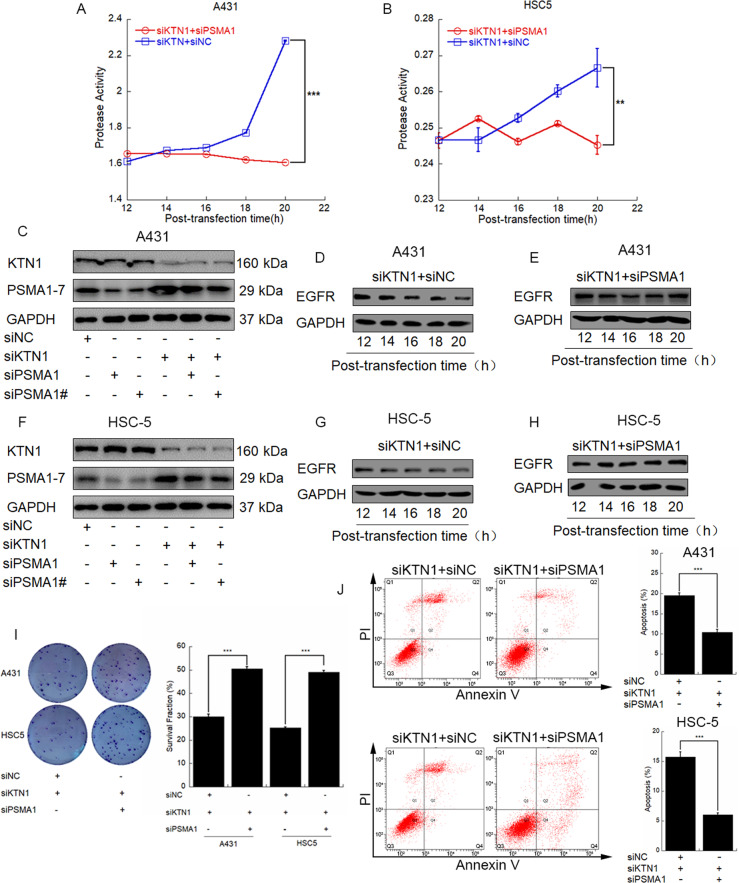
PSMA1 represents a key modulator in KTN1-mediated EGFR degradation. **A**, **B** A431 or HSC-5 cells were transfected with siKTN1 and then harvested to detect protease activity at the indicated times. Data represent the means ± SD. Representative of results from three biological replicates. **C** A431 cells were transfected with siKTN1 and/or siPSMA1/siPSMA1#. Total cell lysates were subjected to immunoblotting with anti-PSMA1-7 and anti-KTN1. Representative of results from three biological replicates. **D**, **E** A431 cells were transfected with siKTN1 or co-transfected with siKTN1 and siPSMA1. Total cell lysates were collected after transfection for the indicated time periods and analyzed by immunoblotting with anti-EGFR. Representative of results from three biological replicates. **F** HSC-5 cells were transfected with siKTN1 and/or siPSMA1/siPSMA1#, and total cell lysates were subjected to immunoblotting with anti-PSMA1 and anti-KTN1. Representative of results from three biological replicates. **G**, **H** HSC-5 cells were transfected with siKTN1 or co-transfected with siKTN1 and siPSMA1. Total cell lysates were collected at the indicated times and analyzed by immunoblotting with anti-EGFR. Representative of results from three biological replicates. **I** A431 or HSC-5 cells were transfected with siKTN1 or co-transfected with siKTN1 and siPSMA1. Total cell lysates were collected for colony formation assay. Survival fractions represent the means ± SD. Representative of results from three biological replicates. **J** A431 or HSC-5 cells were transfected with siKTN1 or co-transfected with siKTN1 and siPSMA1. Total cell lysates were collected for flow cytometry analysis of apoptotic cells. Apoptotic cell ratios represent the means ± SD. Representative of results from three biological replicates.

To determine whether PSMA1 serves as a key modulator of KTN1-mediated EGFR degradation, we next detected protease activity in A431 and HSC-5 cells after co-transfection with siKTN1 and siPSMA1. The protease activity significantly decreased after knockdown of KTN1 and PSMA1 compared to only knockdown of KTN1 (Fig. 4A, B). To verify these results, we detected EGFR levels by western blotting after transfection with siKTN1 and either siNC or siPSMA1. Whereas EGFR protein expression decreased from 12 to 20 h of post-transfection in the siKTN1 + siNC co-transfection group, there was no significant change in EGFR expression in the siKTN1 + siPSMA1 co-transfection group for both A431 (Fig. 4C–E) and HSC-5 (Fig. 4F–H) cells. Furthermore, the survival fraction was increased (Fig. 4I) and the apoptotic rate was decreased (Fig. 4L) after transfection of siKTN1 + siPSMA1 compared to siKTN1 + siNC in both in A431 and HSC-5 cells. These results indicate that PSMA1 serves as a key modulator of EGFR degradation and subsequent effects on tumor cell survival induced by knockdown of KTN1.

### c-MYC is upregulated by KTN1 knockdown and directly binds to the CCDC40 promoter region to transactivate CCDC40

**Fig. 5:**
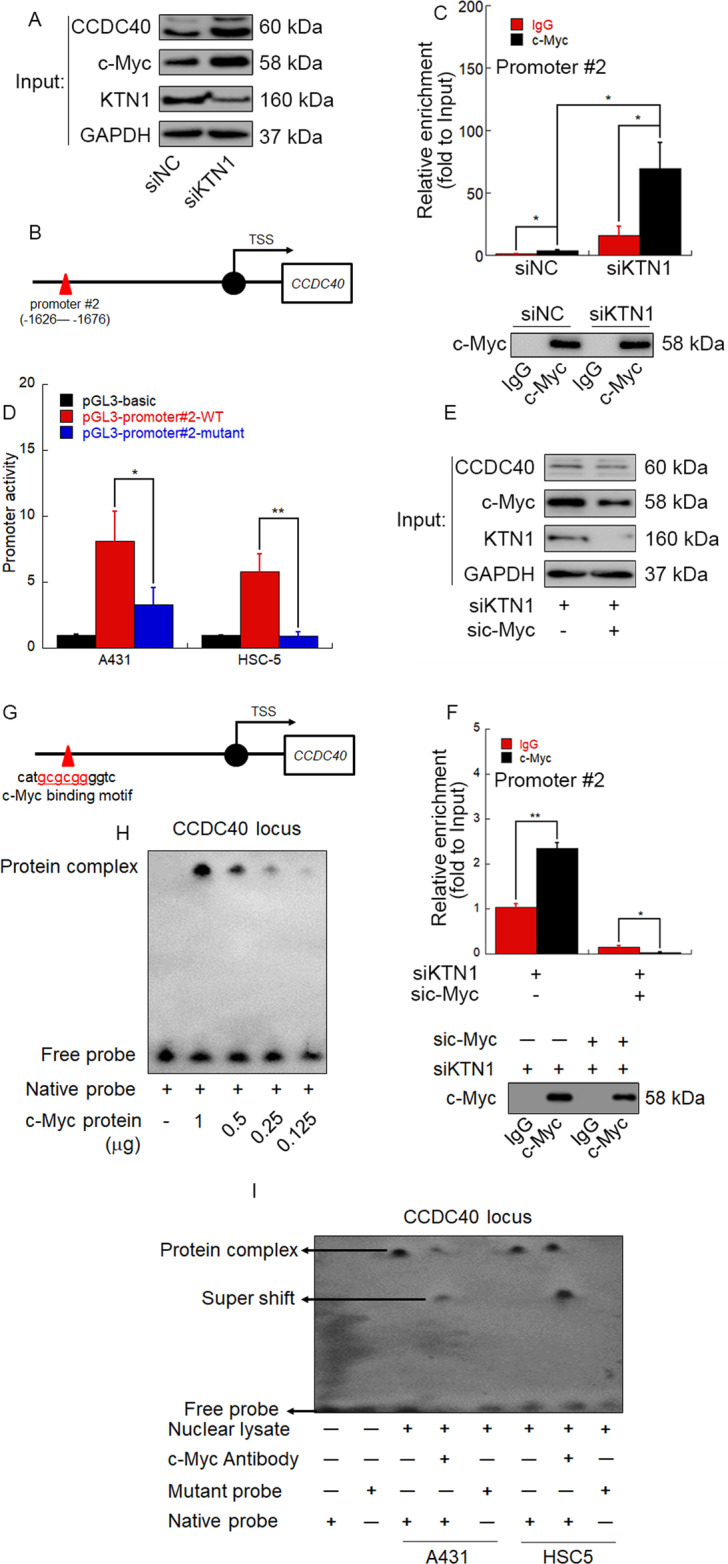
c-MYC is upregulated by KTN1 knockdown and directly binds to the promoter region to transactivate CCDC40. **A** Schematic diagram for the positions of two predicted promoters in the *CCDC40* gene. **B**, **C** A431 cells were transfected with siKTN1. Total cell lysates were subjected to ChIP-qPCR assay and immunoblotting. TSS: transcription start site. qPCR results represent the means ± SD. Representative of results from three biological replicates. **D** Luciferase reporter assay of the predicted promoter #2. Data represent the means ± SD. Representative of results from three biological replicates. **E**, **F** A431 cells were transfected with siKTN1 or co-transfected with siKTN1 and sic-Myc. Total cell lysates were subjected to ChIP-qPCR assay and immunoblotting. qPCR results represent the means ± SD. Representative of results from three biological replicates. **G** Schematic diagram for the predicted interaction site (red underlined bases) of c-Myc (c-Myc binding motif) in *CCDC 40* promoter #2. **H** Electrophoretic mobility shift assay (EMSA) to detect the direct interaction between the c-Myc binding motif and purified c-MYC protein. Representative of results from three biological replicates. **I** Nuclear lysates from A431 and HSC-5 cells were subjected to EMSA to detect the direct interaction between the c-Myc binding motif and nuclear protein. Anti-c-Myc antibody was added for supershift of the binding complex. Representative of results from three biological replicates.

In our previous study, we reported that c-Myc can bind to the promoter region of KTN1 and mediate its transactivation. Therefore, we hypothesized that c-Myc may also act as a transcription factor for CCDC40. To evaluate this possibility, we first detected c-Myc protein levels in cSCC cells after knockdown of KTN1. The results suggest that c-Myc protein levels are increased in A431 and HSC-5 cells transfected with siKTN1 versus siNC (Fig. 5A and Fig. S3A). Next, we entered the 2000nt upstream CDS region of the CCDC40 gene into the Berkeley Drosophila Genome Project web server (https://insitu.fruitfly.org/cgi-bin/ex/insitu.pl) and obtained two candidate promoters (Fig. 5B). We performed chromatin immunoprecipitation (ChIP)-qPCR to determine whether one of these predicted promoters might interact with c-Myc. The predicted promoter #2 was immunoprecipitated by c-Myc antibody. Moreover, enrichment of promoter #2 significantly increased after knockdown of KTN1, which suggests that KTN1 knockdown enhances the c-Myc-mediated transactivation of the CCDC40 promoter #2 in cSCC cells (Fig. 5C and Fig. S3B). To verify that promoter #2 acts as a functional promoter in cSCC cells, we designed WT and mutant luciferase reporter plasmids. Promoter #2 displayed transactivation activity that was reduced by mutation (Fig. 5D). To further confirm a role for c-Myc in promoter #2 transactivation induced by KTN1, we performed ChIP-qPCR in cSCC cells with knockdown of KTN1 and *c-Myc*. The enrichment of promoter#2 was significantly decreased by knockdown of KTN1 and *c-Myc* compared to knockdown of KTN1 alone (Fig. 5E, F and Fig. S3C, D). Notably, promoter #2 is predicted by the rVista 2.0 web tool [32] to contain an E-box sequence (CGCGGG), which serves as a probable binding motif for c-Myc (Fig. 5G). Direct binding of c-Myc at the E-box locus was confirmed by electrophoretic mobility shift assay (EMSA) (Fig. 5H, I). All these findings strongly suggest that c-Myc directly binds to the promoter region of CCDC40 to induce its expression in siKTN1-transfected cells.

### The CCDC40-ADRM1-UCH37 axis plays a crucial role in the deubiquitination of EGFR

**Fig. 6:**
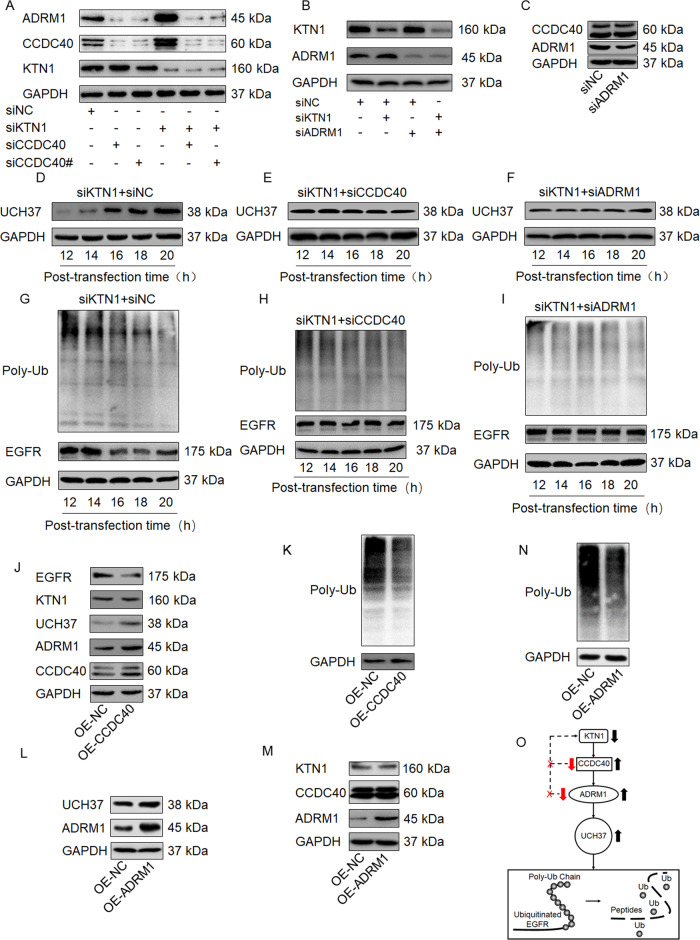
The CCDC40-ADRM1-UCH37 axis plays a crucial role in the deubiquitination of EGFR. **A** A431 cells were transfected with siNC or/and siKTN1 or/and siCCDC40 or/and siCCDC40#. Total cell lysates were subjected to immunoblotting with anti-KTN1, anti-CCDC40, and anti-ADRM1. Representative of results from three biological replicates. **B** A431 cells were transfected with siNC or/and siKTN1 or/and siADRM1. Total cell lysates were subjected to immunoblotting with anti-KTN1 and anti-ADRM1. Representative of results from three biological replicates. **C** A431 cells were transfected with siKTN1 or siADRM1. Total cell lysates were subjected to immunoblotting with anti-ADRM1 and anti-KTN1. Representative of results from three biological replicates. **D**–**I** A431 cells were transfected with siKTN1 alone or in combination with siCCDC40 or siADRM1. Total cell lysates were collected at the indicated times. Total cell lysates were analyzed by immunoblotting with anti-UCH37, anti-EGFR, and anti-poly-ubiquitin. Representative of results from three biological replicates. **J**, **K** A431 cells were transfected with plasmid expressing CCDC40. Total cell lysates were subjected to immunoblotting with anti-CCDC40, anti-ADRM1, anti-UCH37, anti-KTN1, anti-EGFR, and anti-poly-ubiquitin. Representative of results from three biological replicates. **L**–**N** A431 cells were transfected with plasmid expressing ADRM1. Total cell lysates were subjected to immunoblotting with anti-CCDC40, anti-ADRM1, anti-UCH37, anti-KTN1, anti-EGFR, and anti-poly-ubiquitin. Representative of results from three biological replicates. **O** Schematic diagram of the role of the CCDC40-ADRM1-UCH37 axis in deubiquitination of EGFR induced by KTN1 depletion.

Given that CCDC40 and ADRM1 are both upregulated after knockdown of KTN1 in cSCC cells, we sought to explore whether the regulation of CCDC40 and ADRM1 may be interrelated. CCDC40 knockdown decreased ADRM1 protein expression in both A431 (Fig. 6A) and HSC-5 (Fig. S4A) cells, though there was no significant difference in KTN1 protein levels after transfection with siCCDC40 compared to siNC. Conversely, knockdown of ADRM1 had no effect on CCDC40 expression (Fig. 6B, C and Fig. S4B, C). These findings indicate that CCDC40, which is upregulated upon KTN1 knockdown, is an upstream activator of ADRM1.

ADRM1 is known to directly bind to UCH37, which is one of the three principal proteasome-associated deubiquitinating enzymes (DUBs) [33, 34]. To determine whether CCDC40 and ADRM1 affect UCH37 levels induced by knockdown of KTN1, we performed western blotting at five time points from 12 to 20 h after co-transfection with siKTN1 and siADRM1 or siKTN1 and siCCDC40. The UCH37 levels were not significantly affected by co-transfection with either siADRM1 or siCCDC40 (Fig. 6D–F and Fig. S4D–F); however, siCCDC40 and siADRM1 co-transfection each attenuated the siKTN1-mediated reduction in EGFR levels and the siKTN1-mediated decrease in poly-ubiquitin protein levels both in A431 and HSC-5 cells (Fig. 6G–I and Fig. S4G–I). The above results indicate that knockdown of CCDC40 and ADRM1 inhibits deubiquitination without affecting the UCH37 level.

To validate the importance of CCDC40 and ADRM1 in deubiquitination of EGFR, we performed gain-of-function assays. Overexpression of CCDC40 (OE-CCDC40) compared to NC (OE-NC) increased the UCH37 levels and decreased the poly-ubiquitin protein levels in both A431 and HSC-5 cells (Fig. 6J, K and Fig. S4J, K). Furthermore, similar results were observed for ADRM1 overexpression (Fig. 6L–N and Fig. S4L–N), though it had no observable effect on KTN1 expression. These results suggest that the CCDC40-ADRM1-UCH37 regulation axis plays a crucial role in the deubiquitination of EGFR (Fig. 6O), but that effects on UCH37 levels within this axis may be subtle or require high CCDC40 and ADRM1 expression.

### KTN1 and PSMA1 interact with each other and competitively bind ADRM1 via amino acid residues Met1-Ala252

**Fig. 7:**
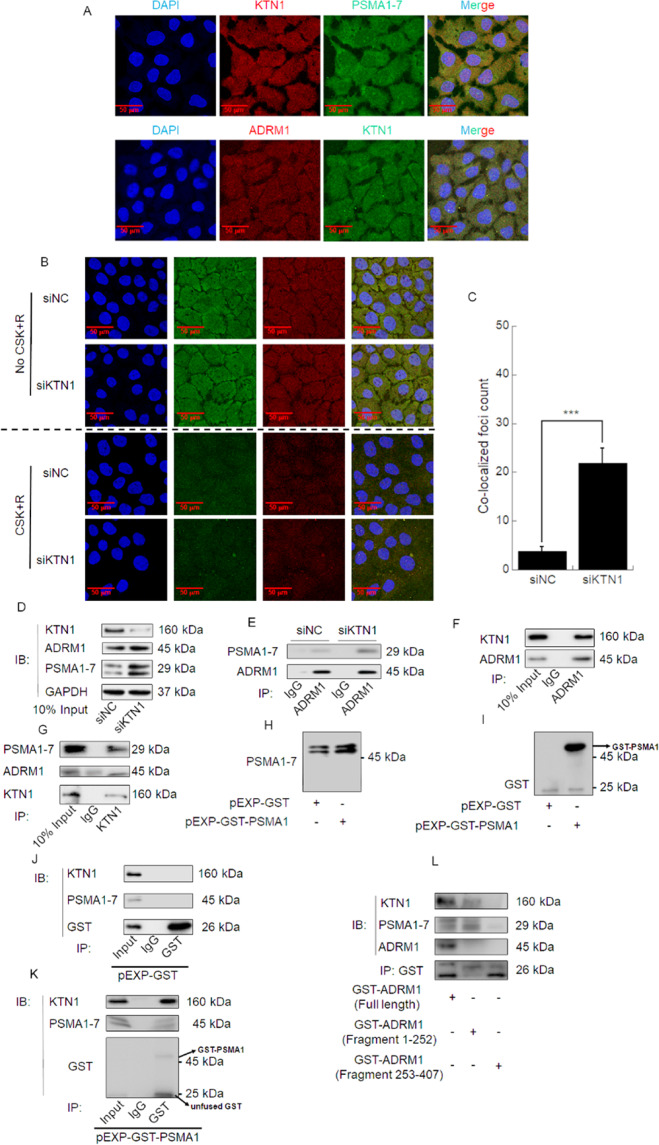
KTN1 and PSMA1 interact with each other and competitively bind ADRM1 at amino acid residues Met1–Ala252 in A431 cells. **A** Confocal immunofluorescent staining images showing co-localization between KTN1 and PSMA1–7 or ADRM1 in A431 cells. Representative of results from three biological replicates. **B**, **C** A431 cells were transfected with siKTN1 or siNC and then were treated with or without CSK buffer. Confocal immunofluorescent staining images show co-localization between PSMA1–7 and ADRM1 in A431 cells. Quantified foci count represents the means ± SD. Representative of results from three biological replicates. **D**, **E** A431 cells were transfected with siKTN1 or siNC. Total cell lysates were subjected to Co-IP and immunoblotting with anti-PSMA1–7, anti-ADRM1, or anti-KTN1. Representative of results from three biological replicates. **F**, **G** A431 cell lysates were subjected to Co-IP analysis. Representative of results from three biological replicates. **H**–**K** A431 cells were transfected with plasmid expressing GST or GST-tagged PSMA1(GST-PSMA1). Representative of results from three biological replicates. Total cell lysates were subjected to Co-IP and immunoblotting with anti-GST, anti-PSMA1–7 or anti-KTN1. Representative of results from three biological replicates. **L** A431 cells were transfected with plasmid expressing GST-tagged ADRM1 full length, GST-tagged ADRM1 1–252 amino acid residues or GST-tagged ADRM1 253–407 amino acid residues. Total cell lysates were subjected to Co-IP and immunoblotting with anti-GST, anti-ADRM1, anti-PSMA1–7, or anti-KTN1. Representative of results from three biological replicates.

To further explore the relationship among KTN1, PSMA1, and ADRM1, we performed immunofluorescent staining. Merged figures showed that KTN1, PSMA1, and ADRM1 co-localize with each other. Moreover, PSMA1 and ADRM1 co-localization foci increased after knockdown of KTN1 compared to NC in A431 and HSC-5 cells (Fig. 7A–C and Fig. S5A–C). These results suggest that KTN1 may competitively inhibit the interaction between PSMA1 and ADRM1.

To verify the interaction among KTN1, PSMA1, and ADRM1, we performed Co-IP assays. The results verify that the interaction between PSMA1 and ADRM1 was strengthened after knockdown of KTN1 (Fig. 7D, E and Fig. S5D, E). We also used an anti-ADRM1 antibody to immunoprecipitate KTN1; and reciprocally, an anti-KTN1 antibody to immunoprecipitate ADRM1, which confirms that KTN1 interacts with ADRM1 (Fig. 7F, G and Fig. S5F, G). As additional confirmation, a GST-PSMA1 plasmid was used to express a GST-tagged PSMA1 fusion protein in A431 and HSC-5 cells (Fig. 7H, I and Fig. S5H, I). Next, an anti-GST antibody was used to immunoprecipitate PSMA1, which also pulled down KTN1 in the immunoblot, thus verifying their interaction (Fig. 7J, K and Fig. S5J, K). Altogether, these results demonstrated that KTN1 interacts with PSMA1 and ADRM1, and that PSMA1 and ADRM1 interact with each other.

ADRM1 has been demonstrated to interact with UCH37 at amino acid residues Ser253-Asp407 [35]. Thus, to further investigate which amino acid residues are involved in its competitive interaction with PSMA1 and KTN1, we used GST-tagged ADRM1 constructs to express GST-tagged ADRM1 fragments (1–252 and 253–407) and GST tagged ADRM1 (full length) fusion protein in A431 and HSC-5 cells. Immunoprecipitation using anti-GST antibody suggests that KTN1 and PSMA1 each bind to ADRM1 via amino acid residues Met1-Ala252 (Fig. 7L and Fig. S5L).

### In vivo function of the EGFR degradation regulatory axis is triggered by KTN1

**Fig. 8:**
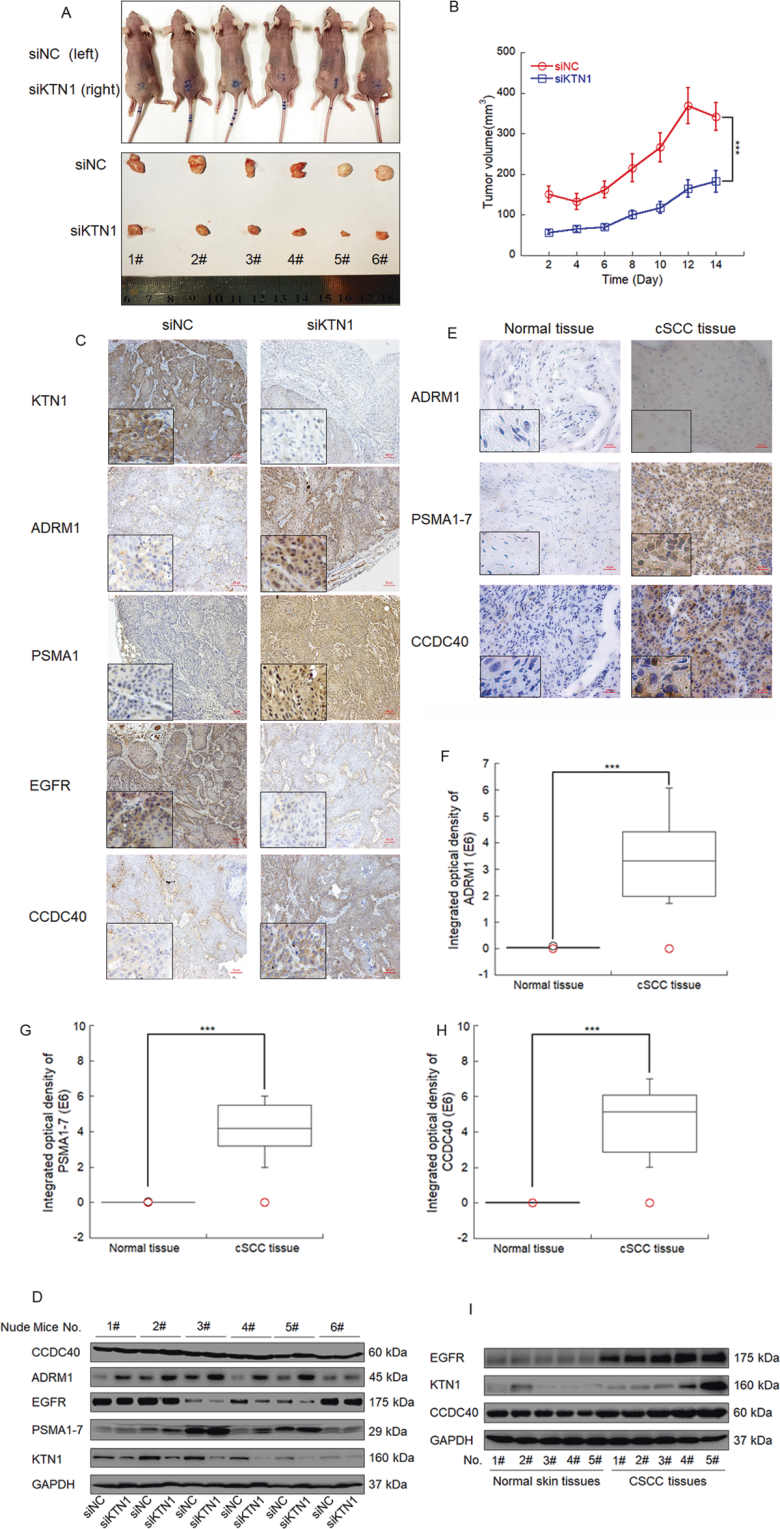
In vivo function of the EGFR degradation regulatory axis triggered by KTN1. **A**, **B** Tumorigenicity of KTN1 depletion and control A431 cells in nude mice. siKTN1-transfected or siNC-transfected A431 cells (0.1 mL; 2 × 10^6^ cells) were injected into different sides of nude mice. Tumor growth was measured every 2 days. Two weeks later, the mice were sacrificed to collect the tumor samples. Photo credit: Ji Ma, Department of Radiation medicine, School of Public Health, Southern Medical University. Tumor volumes represent the means ± SD. **C** Representative photos of immunohistochemistry (IHC) staining with anti-PSMA1–7, anti-ADRM1, anti-EGFR, anti-CCDC40, and anti-KTN1 for the xenograft tumor model. **D** Lysates of the xenografted tumors were subjected to immunoblotting with anti-PSMA1–7, anti-ADRM1, anti-EGFR, anti-CCDC40, or anti-KTN1. **E**–**H** Representative photos and integrated optical densities of immunohistochemistry (IHC) staining with anti-PSMA1–7, anti-ADRM1, and anti-CCDC40 for normal human tissues and human cSCC tissues. Data represent the means ± SD. **I** Lysates of normal human tissues and human cSCC tissues were subjected to immunoblotting with anti-PSMA1-7, anti-ADRM1, or anti-CCDC40.

To solidify the function of the EGFR degradation regulatory axis triggered by KTN1 in an in vivo model system, we employed the mouse xenograft model. We injected cSCC cells expressing siNC on the left flanks and siKTN1 on the right flanks or the same mouse. The tumor volumes of the siKTN1 tumors were significantly smaller than those of the siNC tumors (Fig. 8A, B). Immunohistochemistry and Western blot assays verified that the expression of PSMA1, CCDC40, and ADRM1 in the xenografts were increased after knockdown of KTN1 compared to NC, and that the expression of EGFR was decreased (Fig. 8C, D).

To verify the central role of PSMA1 in the xenograft model, we repeated the injections with siKTN1 + siNC-co-transfected cells in the left flanks and siKTN1 + siPSMA1-co-transfected cells in the right flanks. The tumor volumes of the siKTN1 + siPSMA1 co-transfected tumors were significantly larger than those of the siKTN1 + siNC co-transfected tumors (Fig. S6A, B). Furthermore, immunohistochemistry and Western blot assays demonstrated that the expression of EGFR was increased in the siKTN1 + siPSMA1 co-transfection group relative to the siKTN + siNC cotransfection group (Fig. S6C, D).

Finally, to verify the relevance of this regulatory pathway to human cSCC, we obtained clinical samples of matched normal skin tissues and cSCC tissues. Immunohistochemical analysis revealed that ADRM1, PSMA1, and CCDC40 were all expressed more highly in cSCC tissues (Fig. 8E–H). Furthermore, the general pattern of very high expression of EGFR and co-elevated expression of CCDC40 and KTN1 in cSCC tissues was confirmed (Fig. 8E–I). These findings suggest a model by which reduction in KTN1 expression in cSCC may promote EGFR degradation via the UPS (Fig. S7A, B).

## Discussion

In this study, we demonstrate a critical role for the UPS in mediating the expression of EGFR in cSCC. While misfolded protein accumulation causes ER stress activation [36], the unfolded protein response (UPR) is a vital cellular signaling pathway for alleviation of ER stress to maintain homeostasis [37]. UPS activation, and especially the 20S proteasome, has been reported to alleviate ER stress [38, 39]. Once ER transmembrane receptors ATF6, PERK and IRE1α are activated, TRAF2 is recruited to the ER membrane by IRE1α to trigger NF-κB signaling [40]. A series of responses results in important cellular events, such as apoptosis and autophagy [41, 42].

Notably, we demonstrated a novel mechanism by which KTN1 deficiency regulates EGFR degradation via UPS activation. KTN1 is accumulated in integrin-based adhesion complexes (IAC) in the endoplasmic reticulum (ER) lumen and membrane to serve in kinesin-driven vesicle motility [23, 43]. In our previous study, we performed transcriptomic sequencing (RNA-Seq) and demonstrated that KTN1 is downregulated after knockdown of MALAT1 in cSCC cell lines. KTN1 has been shown to serve as a mediator for MALAT1 regulation of EGFR protein rather than RNA expression [18]. Our results demonstrate that KTN1 depletion not only increases protein expression of PSMA1 but maintains the stability of proteasome holoenzyme, which is supported by the competitive binding of KTN1 to the 1–254 amino acids site of ADRM1. We also demonstrated that KTN1 depletion activates transcription of CCDC40, which increases the expression of ADRM1, resulting in EGFR deubiquitination via UCH37. These molecular events induce EGFR degradation and anti-tumor activity in cSCC. We further confirmed that knockdown of *KTN1* promotes EGFR degradation via the UPS, which is carried out by PSMA1 in cSCC. Our findings thus reveal a new regulatory axis of the ER constitutive protein KTN1 in promoting EGFR degradation.

C-myc is a general transcription factor for many genes in eukaryotic cells. Our previous research revealed that c-myc serves as a transcription factor of KTN1 to activate downstream regulation [18]. In this study, we further demonstrated that knockdown of KTN1 increases the expression of c-myc, which forms a negative feedback regulator. Moreover, we demonstrated for the first time that c-myc interacts with the CCDC40 promoter and activates its expression. In this study, CCDC40 was identified as the most highly upregulated protein in differential expression studies of *KTN1* knockdown. CCDC40 participates in the assembly of the dynein regulatory complex and the inner dynein arm complex, both of which play crucial roles in ciliary beat regulation of 96 nanometer (nm) repeat length and arrangement of components in cilia and flagella. Aberrant function of CCDC40 has been reported to cause primary ciliary dyskinesia [44]. Furthermore, CCDC40 has been shown to be essential for left-right axis formation in tissue development, which may be related to congenital heart disease [45]. In this study, we demonstrate that CCDC40 is upregulated by KTN1 depletion, which triggers the CCDC40-ADRM1-UCH37 regulatory axis as an anti-tumor pathway in cSCC. In this pathway, CCDC40 induces the expression of ADRM1 to recruit UCH37, which accelerates the ubiquitin cycle and promotes UPS for EGFR degradation. This the first demonstration that this axis functions as a tumor-related regulatory pathway, which provides insight into additional functions of CCDC40. Thus, our findings may reveal a novel connection between dynein and tumorigenesis.

The proteasome is a multi-subunit complex that lies at the core in the degradation of selectively ubiquitylated proteins. The proteosome has been reported to possess three protease activities—caspase-like activity, trypsin-like activity, and chymotrypsin-like activity—which are mediated by PSMB1, PSMB2, and PSMB5 in the 20S proteasome [46]. PSMA1, an α-ring component involved in the proteolytic degradation of most intracellular proteins, is downregulated by autophagic lysosome-induced TRAF6 degradation to inhibit the proteasome and result in apoptosis in acute myeloid leukemia [47]. The functions of additional 20S α-subunits have not been well documented; however, our results demonstrate that PSMA1 is upregulated upon KTN1 deficit, which results in degradation of excess EGFR to inhibit cSCC. The 20S CP binds to 19S RPs to form the 26S (single capped) or 30S (double capped) holoenzyme for proteolysis [48]. The 19S RP consists of a lid, which contains non-ATPase proteins (including ADRM1); and a base, which contains AAA + ATPases as the molecular motor of the proteasome [49]. The deubiquitinating enzyme PSMD14 is a non-ATPase component of the lid structure. PSMD14 and another deubiquitinating enzyme family member, UCH37, are recruited and activated by ADRM1. ADRM1 is recognized to bind ubiquitin through its pleckstrin-like receptor for ubiquitin (Pru) domain at amino acid residues Tyr22-Asn130 [50]. In addition, PSMD14 has a UCH37 binding domain, which encompasses amino acid residues Ser253-Asp407 [35]. Our findings reveal that KTN1 and 20S CP competitively interact via amino acid residues Met1-Ala252 of PSMD14 rather than the UCH37 binding domain, suggesting a distinct level of regulation of proteosomal processing mediated by PSMD1 interaction.

Proteasome not only serve as an executor for UPS to degrade massive proteins, but also as a stimulus for the important pathways of reaction to drive cell fate decision. Accumulating studies have been reported vital molecular mechanisms in regulating tumor [51]. Further, due to high energy metabolic rates, reprogrammed and genomic instability, cancer cells are exposed to considerable protein overload, which are easier to triggered UPS [52]. Lots of results indicates that increased protease activity could promote cancer cell growth. It is reported that Poly (rC) binding protein 2 (PCBP2) directly interacted with both tribbles homolog 2 (TRIB2) and PSMB5, and TRIB2 plays a critical role by modulating PSMB5 activity in reducing Ub flux, this interaction was found to maintain the viability of the liver cancer cells and promote tumor growth [53]. If the substrate are the growth factor relative proteins, protease activity strengthen also will led to tumor inhibition. As a study prove that circPABPC1 (polyadenylate-binding protein 1) inhibits cell adhesion and migration through directly links ITGB1 to the 26S proteasome for degradation itself [54].

In summary, we revealed a new axis by which KTN1 triggers EGFR protein degradation via the UPS. Our study provides mechanistic insight into the functions of KTN1 in protein degradation and the relationship between protein degradation, ER stress and the UPS. These findings suggest a potential direction for EGFR-targeted therapy for cSCC treatment. Further investigation into the function of KTN1 in ER is warranted and may demonstrate new promising regulatory networks for ER stress in tumorigenesis and cancer treatment.

## Materials and methods

### Cell culture

Human benign epidermal keratinocyte HaCaT cells were obtained from the China Center for Type Culture Collection (China). The human cutaneous squamous cell carcinoma cell lines A431, HSC1, HSC5, and SCL-1 were obtained from HonSun Biological Co. Ltd. (China). All cells were grown in Dulbecco Modified Eagle Medium supplemented with 10% fetal bovine serum and 1% Penicillin-Streptomycin Solution (Gibco, USA), at 37 °C in 5% CO_2_ in a humidified incubator (Thermo Scientific, USA). CHX was purchased from Beyotime (Beyotime Biotechnology, China) and was used at a concentration of 50 μg/mL. MG-132 was purchased from Selleck (Selleckchem, USA) and was used at concentration of 10 μmol/mL.

### Plasmids and siRNA

Human ADRM1, CCDC40, GST, GST-ADRM1-full length, GST-ADRM1-fragment 1, GST-ADRM1-fragment 2, and GST-PSMA1 expression plasmids were constructed by Sangon Biotech (Sangon Biotech, China). pGL3-basic, pGL3-promoter#2-WT (ATGCGCGGGGTC), and pGL3-promoter#2-mutant (CGTATCTTTAGC) plasmids were also constructed by Sangon Biotech (Sangon Biotech, China). Supplementary Table 1 listed siRNAs used to silence target genes by transfection with Lipofectamine 2000 (Life Technologies–Invitrogen, USA), according to the manufacturer’s instructions (Table S2).

The proteins in each sample were reduced with dithiothreitol (DTT) and then alkylated with iodoacetamide. The iTRAQ assay was performed at Shanghai Applied Protein Technology Co., Ltd. Differentially expressed proteins identified by iTRAQ were analyzed by hierarchical clustering, gene oncology, KEGG, and protein–protein interaction analyses. Details are provided in Supplementary Materials (Table S1).

### Animal experiments

Five-week-old nude mice were randomly divided into two groups. A431 cells were transfected with siNC, siKTN1, siKTN1 + siNC, or siKTN1 + siPSMA1. A total of 5 × 10^6^ cells were injected subcutaneously into the flanks of mice (siNC vs. siKTN1, *n* = 8, siKTN1 + siNC vs. siKTN1 + siPSMA1, *n* = 8). When the tumor volumes reached 100 mm^3^, they were injected with corresponding siRNA at 2 days intervals. Tumor volumes were measured every 2 days for 2 weeks using a caliper. All tumor volume data were normalized to the volumes obtained just before injection. The mice were maintained in the SPF Animal Laboratory at Southern Medical University. All animal studies were reviewed and approved by the Southern Medical University Institutional Animal Care and Use Committee.

### Immunohistochemistry (IHC)

Thirteen samples of surgically removed cSCC and ten paired samples of normal skin tissues were obtained from the Department of Surgery of Nanfang Hospital, Southern Medical University. The Ethics Committee of the Southern Medical University, China, approved our experimental protocols, and donors provided written informed consent. Immunohistochemistry staining was performed at Guangzhou Hypathology Co. Scoring of tissue slides was performed independently by two investigators using the 12-tier scoring system in which the IHC score was calculated based on integrated optical density analyzed by Image J (NIH, USA).

### Statistics

Data are expressed as mean ± standard deviation (SD). All statistical analyses were performed with SPSS software (Version 22.0; Abbott Laboratories, Chicago, IL, USA). Statistical comparisons between two groups were carried out using the Student’s *t*-test. *P* < 0.05 was considered statistically significant (NS for not significant, *P < 0.05; **P < 0.01, ***P < 0.001). At least three independent experiments were performed in all cases.

## Supplementary information

Supplementary Materials

## Data Availability

All data needed to evaluate the conclusions of the paper are presented in the paper and/or Supplementary Materials. Additional data related to this paper may be requested from the authors.
