# Supplementary material for: Kinectin1 depletion promotes EGFR degradation via the ubiquitin-proteosome system in cutaneous squamous cell carcinoma
Source: Cell Death Dis. 2021 Oct 23;12(11):995. doi: 10.1038/s41419-021-04276-5 (PMC8542041; doi:10.1038/s41419-021-04276-5)
Supplement: Supplementary file 1 — Supplementary Materials [file 41419_2021_4276_MOESM1_ESM.docx]

**Kineticin1 depletion promotes EGFR degradation via the ubiquitin-proteosome system in cutaneous squamous cell carcinoma**

Ji Ma,^1^† Shudong Ma,^2^† Ying Zhang,^1^† Yi Shen,^1^ Lei Huang,^3^ Tianhao Lu,^1^ Lu Wang,^1^ Yunhan Wen,^1^ Zhenhua Ding^1^*

^1^ Department of Radiation Medicine, Guangdong Provincial Key Laboratry of Tropical Disease Research, School of Public Health, Southern Medical University, Guangzhou 510515, China.

^2^ Department of Oncology, Nanfang Hospital, Southern Mediacal University, Guangzhou 510515, China.

^3^ Department of Burn, Nanfang Hospital, Southern Mediacal University, Guangzhou 510515, China.

† These authors contributed equally to this work.

* Corresponding author. Email: [dingzh@smu.edu.cn](mailto:dingzh@smu.edu.cn)

**Supplementary Materials**

**MATERIALS AND METHODS**

**RNA extraction and quantitative real-time RCR**

Total RNA was extracted in TRIzol Reagent (Invitrogen, CA, USA) according to the protocol provided by the manufacture. The concentrations of extracted RNAs were determined by spectrophotometry (Nanodrop-2000, Thermo Scientific, NC, USA). Total RNA was reverse-transcribed using the PrimeScript RT Reagent Kit (Takara, Kusatsu, Shiga, Japan). Finally, SYBR Green I-based real-time PCR was performed using TB Green Premix Ex Taq II (Takara, Kusatsu, Shiga, Japan) on the Life Technologies system (QuantStudio 6, NY, USA). Real-time PCR reactions (20 μL total volume) were performed in triplicate using the following program: 2 min at 95°C, followed by 40 thermal cycles of 30 s at 95°C and 40 s at 62°C, and a final extension of 1 min at 72°C. Amplified PCR products were quantified and normalized with GAPDH, and the fold change of mRNA expression was calculated by the 2^-ΔΔCT^method. Primers used in this study are listed in Supplementary Table 2.

**Immunofluorescence**

For PSMA1-7 and ADRM1 co-localization, cells with different treatments were seeded on coverslips, washed with PBS three times and incubated twice for 3 minutes at room temperature with CSK buffer (10 mM Pipes, PH=7.0, 100 mM NaCl, 300 mM sucrose, 3 mM MgCl2) containing 0.7% Triton X-100 and 0.3 mg/ml RNase A (CSK+R). The cells were then fixed with 2% paraformaldehyde in PBS for 15minutes, permeabilized with 0.5% Triton solution for 5 min at 4°C, and blocked with dried skim milk. Subsequently, the cells were incubated with anti-Proteasome 20S alpha (PSMA1-7) / anti-ADRM1 for 2h at room temperature. The cells were washed five times with PBST (Tween-20, 0.1%), incubated with secondary antibody conjugated with Alex Fluor 555 and Alex Fluor 488 for 1h and washed again five times with PBST. The cells were mounted using prolong Gold antifade reagent with DAPI (Life Technology, USA). Finally, the cells were observed under a confocal microscopy (Zeiss, Germany).

For PSMA1-7 and KTN1 co-localization, cells with different treatments were seeded on coverslips and fixed with 4% paraformaldehyde in PBS for 15 m at room temperature. The cells were then permeabilized with 0.5% Triton solution for 5 min at 4°C and blocked with dried skimmed milk. Next, the cells were incubated with anti- Proteasome 20S alpha (PSMA1-7) / anti-Kinectin 1 for 2h at room temperature, washed five times with PBST (Tween-20, 0.1%), incubated with secondary antibody conjugated with Alex Fluor 555 and Alex Fluor 488 for 1 h, and washed again five times with PBST. The cells were then mounted and observed by confocal microscopy as described above.

**Clonogenic assay**

A431 and HSC5 cells were cultured in 60 mm cell culture dishes, seeded at 5×10^5^ per dish. After 24h, the cells were transfected with siKTN1, siNC / siKTN1+ siPSMA1 or siKTN1+ siNC. The cells were harvested and seeded at 100 cells per dish for the siNC and siKTN1 + siPSMA1 groups, or 200 cells per dish for the siKTN1 and siKTN1 + siNC groups. All assays were performed in triplicate. After 14 days of culture, the cells were fixed in 75% ethanol and stained with crystal violet solution.

**Protein isolation and immunoblotting**

For Western blotting assays, cells were harvested and lysed using RIPA buffer (Beyotime Biotechnology, China). Samples were sonicated and centrifuged at 12,000 g for 15 min at 4°C. The concentrations of total protein were determined by using the BCA Assay Kit (Beyotime Biotechnology, China). Samples were then denatured at 100°C for 5 min. Total proteins were separated by 8% SDS-PAGE and transferred to Immobilon®- P membranes (Millipore, Billerica, MA, USA). The membranes were blocked with 5% nonfat milk powder in Tris-buffered saline (pH 7.5) and hybridized overnight with primary antibodies against the following proteins: GAPDH (Santa Cruz, USA), KTN1, ADRM1, EGFR, Ubiquitin, GST (Cell signaling technology, USA), Proteasome 20S alpha (PSMA1-7), UCH37, and CCDC40 (Abcam, USA). After washing, the membranes were hybridized with secondary antibodies conjugated with horseradish peroxidase (Beyotime biotechnology, China) for 1 h. The blots were visualized with an ECL kit (Millipore, Billerica, MA, USA). Grey value was analyzed by Image J (NIH, USA).

**Co-immunoprecipitation**

A431 and HSC5 cells were collected by centrifugation at 2667 rpm (700 g) at 4°C for 15 min in cold PBS. The cell pellets were resuspended in 1 mL of ice-cold IP lysis buffer (Beyotime biotechnology, China), incubated on ice for 5 min and centrifuged at 11495 rpm (13000 g) at 4°C for 5 min. The supernatants were stored at -80°C for use in precipitation experiments. Protein A/G magnetic beads (Bimake, USA) were washed in IP lysis buffer 5 times and then resuspended in 140 μL IP lysis buffer with 1 μg primary antibodies for respective target proteins or IgG. After overnight incubation, the supernatants were discarded, and the beads were washed 5 times in IP lysis buffer. Cell lysates were thawed and incubated with the beads overnight. The supernatants were then discarded, and the beads were washed 5 times in IP lysis buffer. Finally, 160 μL IP lysis buffer and 40 μL 5× loading buffer were added, and the samples were denatured at 100°C for 5 min. Cell lysates were diluted 10-fold in IP lysis buffer, which was used as input. Western blots were used to detect the expression of target proteins with antibodies against the following proteins: EGFR, Ubiquitin, KTN1, ADRM1, GST (Cell signaling technology, USA), and Proteasome 20S alpha (PSMA1-7) (Abcam, USA).

**Cell apoptosis detection**

A431 and HSC5 cells were harvested in 0.25% trypsin solution without EDTA. The cells were washed twice in PBS buffer. The eBioscience™ Annexin V-FITC Apoptosis Detection Kit (Invitrogen, USA) was used to detect cell apoptosis. Procedures were carried out according to the manufacturer’s protocol, and the fluorescence was measured with a FACScan flow cytometer (FACScan, Becton Dickinson).

**Cell protease activity detection**

A431 and HSC5 cells were harvested and lysed in resolving buffer (90 mM Tris base, 90 mM boric acid, 5 mM MgCl2, 0.5 mM EDTA). The Protease Fluorescent Detection Kit (Sigma-Aldrich, USA) was used to detect cell protease activity. Cells were processed according to the manufacturer’s instructions.

**iTRAQ analysis**

The proteins in each sample were reduced with dithiothreitol (DTT) and then alkylated with iodoacetamide. The iTRAQ assay was performed at Shanghai Applied Protein Technology Co., Ltd. Differentially expressed proteins identified by iTRAQ were analyzed by hierarchical clustering, gene oncology, KEGG and protein-protein interaction analyses. Details are provided in Supplementary Materials.

**ChIP-qPCR analysis**

ChIP experiments were performed as recommended by the EZ ChIP^TM^ Chromatin Immunoprecipitation kit manual (Cat. no. 17-371, Merck Millipore, USA) using 5 μg antibody against c-MYC (Cell signaling technology, USA) or isotype IgG (Merck Millipore, USA) as a negative control. Relative enrich of c-MYC at the CCDC40 locus was quantified by qRT-PCR. The primers used for this assay are listed in Supporting Information Table 2.

**Electrophoretic mobility shift assay (EMSA)**

EMSA experiments were carried out using a forward strand 5’ biotin-labelled-dsDNA (Gzscbio, China) and the LightShift™ Chemiluminescent RNA EMSA Kit (Cat. no. 20158, Thermo Scientific, NC, USA). A 50 nM dsDNA probe containing the locus motif at the CCDC40 promoter and a 50 nM dsDNA mutant probe in the locus motif at the CCDC40 promoter were tested for binding with purified c-MYC protein (Cat. no. ab169901, Abcam). Supershift assays were performed by incubating c-MYC antibody with the above locus motif-containing probe and nuclear protein and detecting the formation of reduced mobility band.

**Luciferase reporter assay**

To construct plasmids for detecting the CCDC40 promoter activity, wild-type DNA fragments and mutant fragments were chemically cloned from the predicted human CCDC40 promoter#2 regions and inserted into a pGL3 vector (Sangon, China). A431 and HSC-5 cells were transfected with Renilla luciferase plasmid and pGL3-basic, pGL3-promoter#2-WT or pGL3-promoter#2-Mutant plasmids using Lipofectamine 2000 (Invitrogen, CA, USA). After 48 h, luciferase activity was measured by the Dual-Luciferase Reporter Assay Kit (TransGen Biotech, China).

**Supplementary Figures**


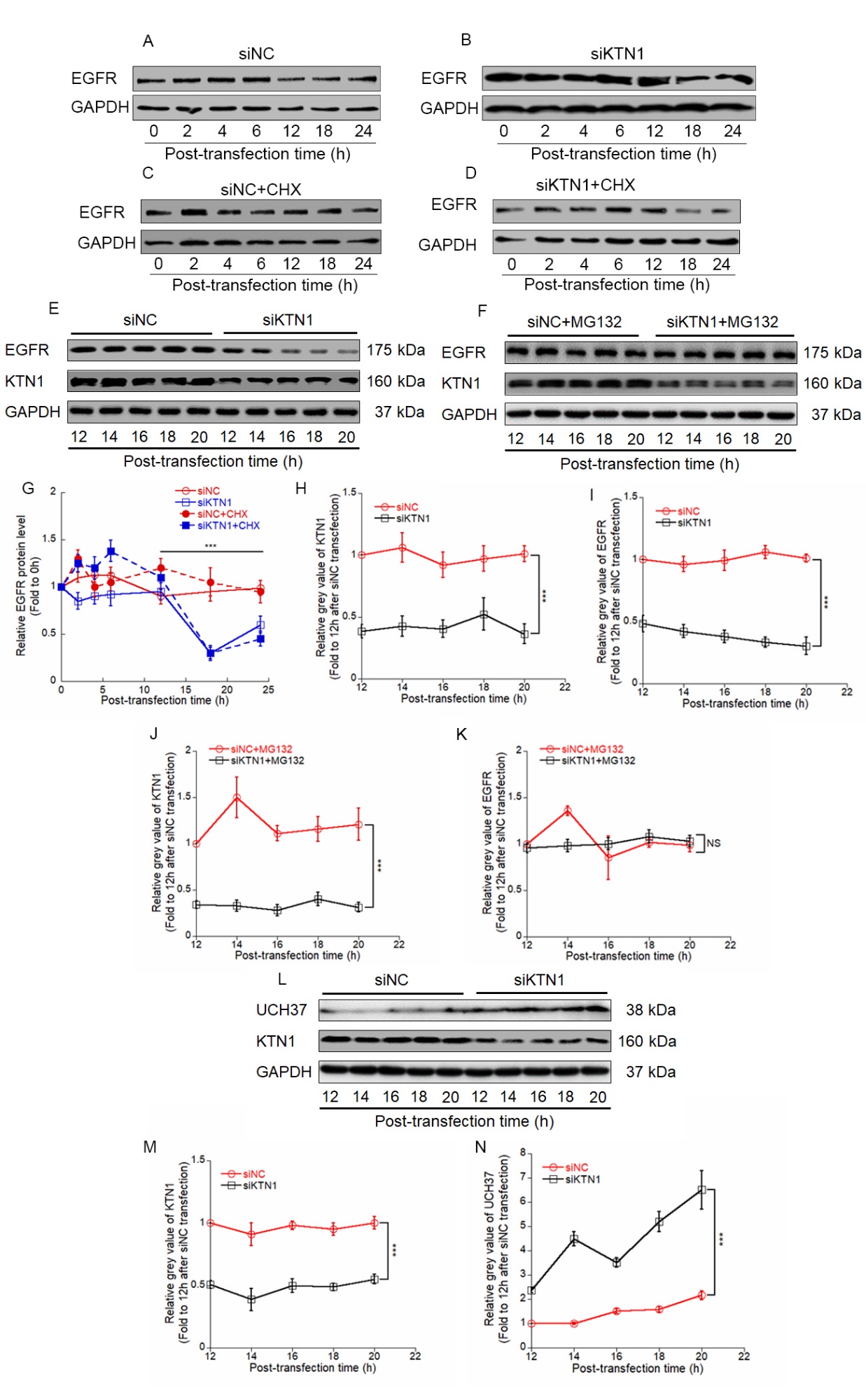


**Fig. S1. KTN1 regulates EGFR degradation via the UPS.**

(**A** and **B**) A431 cells were transfected with siNC or siKTN1 and collected at the indicated times. Total cell lysates were analyzed by immunoblotting with anti-KTN1 and anti-EGFR antibodies. Representative of results from 3 biological replicates. (**C** and **D**) 500nM Cycloheximide (CHX) was added to siNC- or siKTN1-transfected A431 cells and collected at the indicated times. Total cell lysates were analyzed by immunoblotting with anti-KTN1 and anti-EGFR antibodies. Representative of results from 3 biological replicates. (**E**) HSC-5 cells were transfected with siNC or siKTN1 and collected after transfection for the indicated time periods. Total cell lysates were analyzed by immunoblotting with anti-KTN1 and anti-EGFR antibodies. Representative of results from 3 biological replicates. (**F**) 10 μM MG132 was added to siNC- or siKTN1-transfected HSC-5 cells, which were collected after transfection for the indicated time periods. Total cell lysates were analyzed by immunoblotting with anti-KTN1 and anti-EGFR antibodies. Representative of results from 3 biological replicates. (**G** to **K**) Grey value was analyzed by image J and to calculate Relative KTN1 or EGFR protein level. Quantified grey values represent the means ± SD. Representative of results from 3 biological replicates. (**L**) HSC-5 cells were transfected with siNC or siKTN1 and collected at the indicated times. Total cell lysates were analyzed by immunoblotting with anti-UCH37 antibody. Representative of results from 3 biological replicates. (**L and M**) Grey value was analyzed by image J and to calculate Relative KTN1 or UCH37 protein level. Quantified grey values represent the means ± SD. Representative of results from 3 biological replicates.
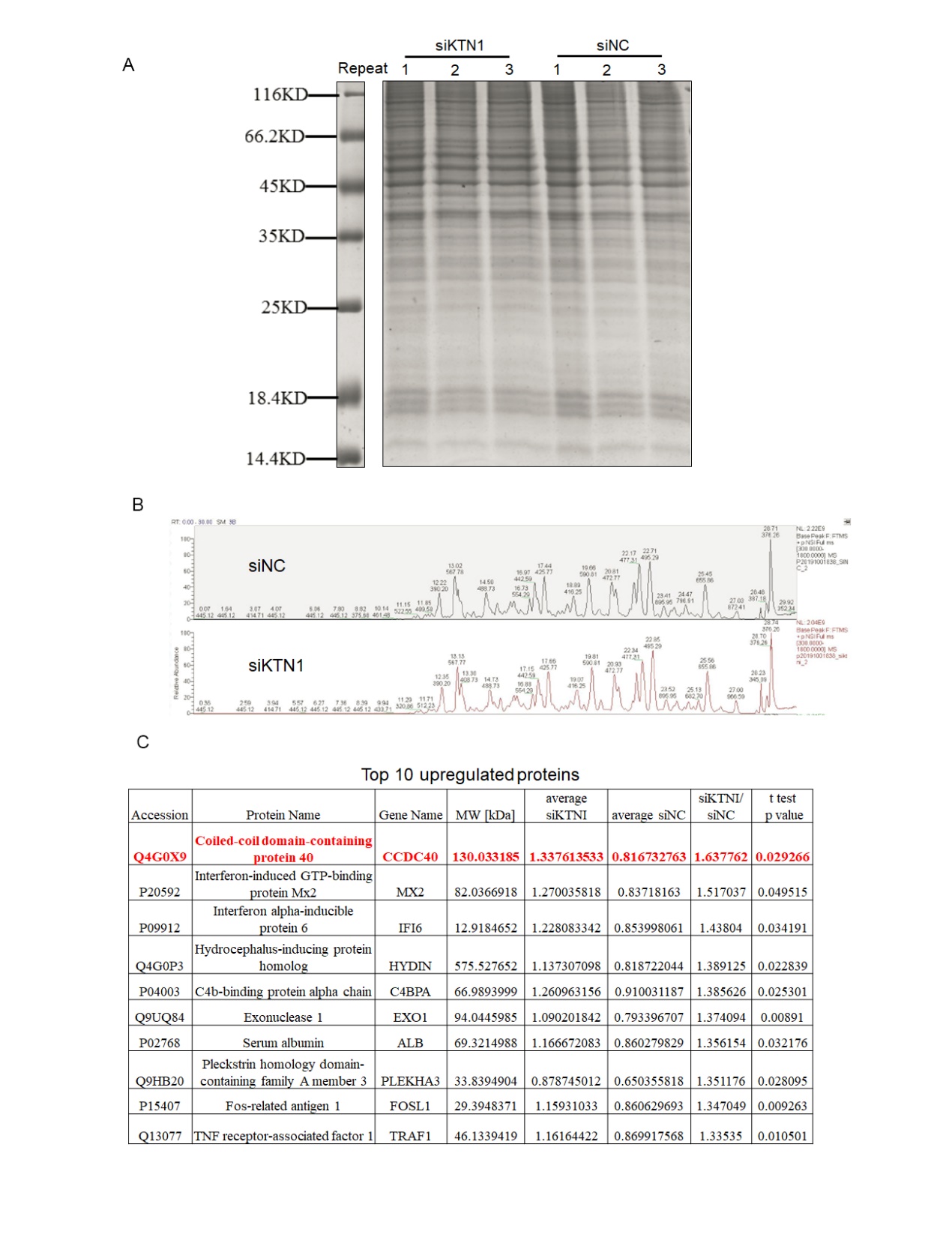


**Fig. S2. CCDC40, PSMA1, and ADRM1 are upregulated by knockdown of *KTN1.***

(**A**) The top 10 upregulated proteins are shown. (**B**) A431 cells were transfected with siNC, siKTN1 or siEGFR in triplicate. Total cell lysates were analyzed by immunoblotting. Representative of results from 3 biological replicates. (**C**) Basepeak for siNC or siKTN1 transfected A431 cell lysates detected by LC-MS/MS.

**
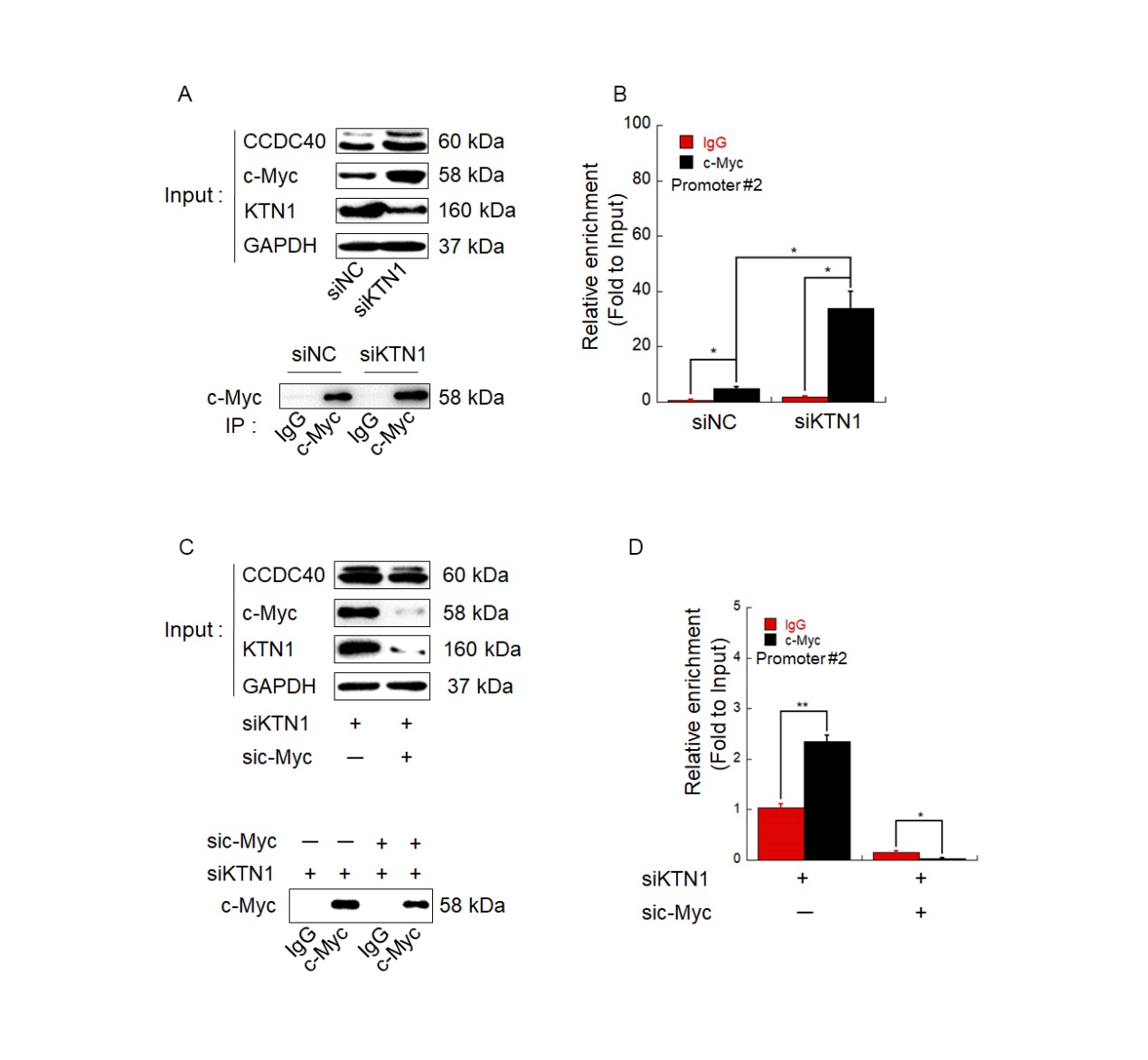
**

**Fig. S3. c-MYC is upregulated by KTN1 knockdown and directly binds and transactivates the promoter region of CCDC40 in HSC-5 cells.**

(**A** and **B**) HSC-5 cells were transfected with siKTN1. Total cell lysates were subjected to ChIP-qPCR assay and immunoblotting. qPCR results represent the means ± SD. Representative of results from 3 biological replicates. (**C** and **D**) HSC-5 cells were transfected with siKTN1 or co-transfected with siKTN1 and sic-Myc, Total cell lysates were subjected to ChIP-qPCR assay and immunoblotting. qPCR results represent the means ± SD. Representative of results from 3 biological replicates.


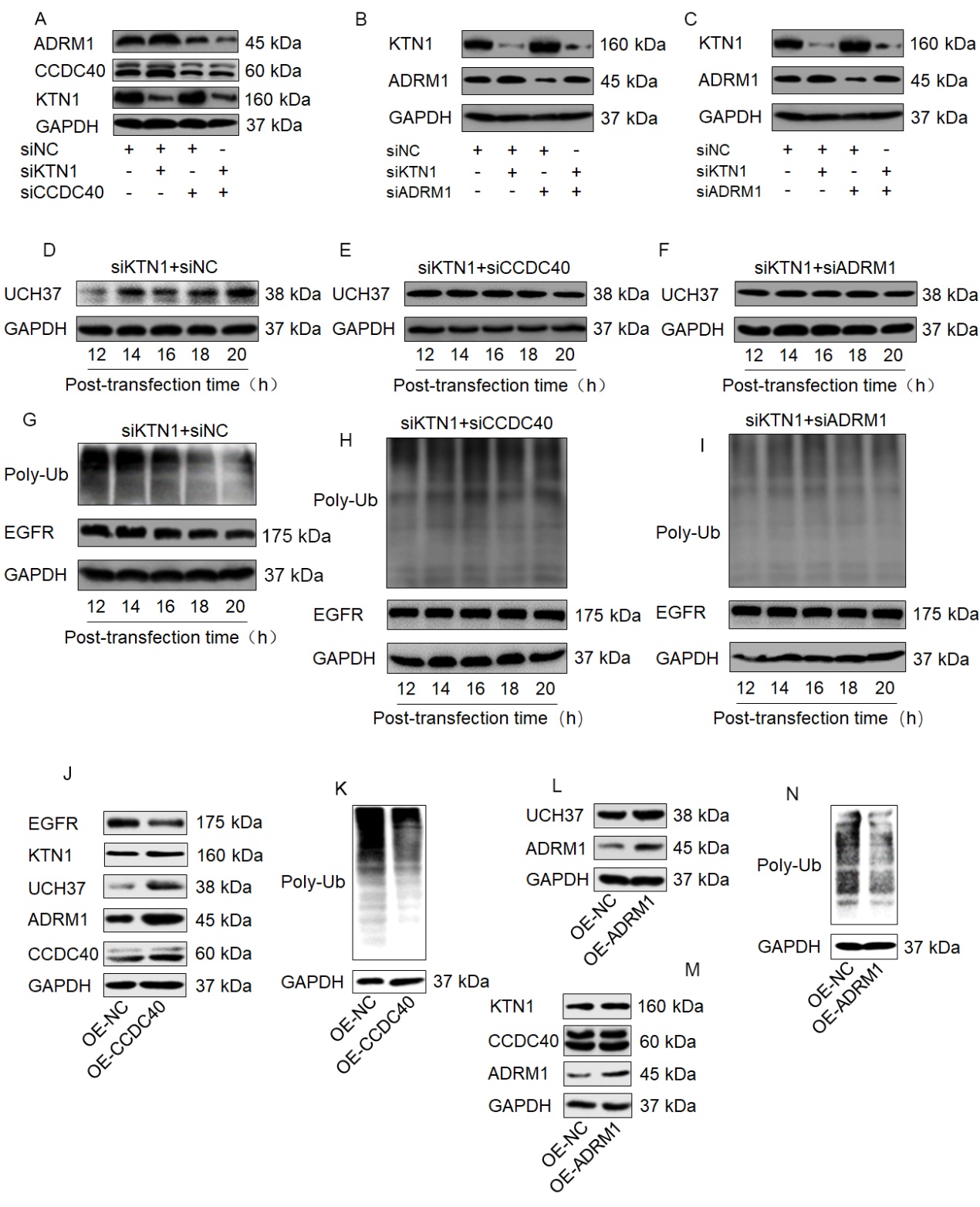


**Fig. S4. The CCDC40-ADRM1-UCH37 axis plays a crucial role in deubiquitination of EGFR.**

(**A**) HSC-5 cells were transfected with siNC or/and siKTN1 or/and siCCDC40 or/and siCCDC40#. Total cell lysates were subjected to immunoblotting with anti-KTN1, anti-CCDC40 and anti-ADRM1. Representative of results from 3 biological replicates. (**B**) HSC-5 cells were transfected with siNC or/and siKTN1 or/and siADRM1. Total cell lysates were subjected to immunoblotting with anti-KTN1 and anti-ADRM1. Representative of results from 3 biological replicates. (**C**) HSC-5 cells were transfected with siKTN1 or siADRM1. Total cell lysates were subjected to immunoblotting with anti-ADRM1 and anti-KTN1. Representative of results from 3 biological replicates. (**D** to **I**) HSC-5 cells were transfected with siKTN1 alone or in combination with siCCDC40 or siADRM1. Total cell lysates were collected at the indicated times post-transfection. Total cell lysates were analyzed by immunoblotting with anti-UCH37, anti-EGFR and anti-poly-ubiquitin. Representative of results from 3 biological replicates. (**J** and **K**) HSC-5 cells were transfected with plasmid expressing CCDC40. Total cell lysates were subjected to immunoblotting with anti-CCDC40, anti-ADRM1, anti-UCH37, anti-KTN1, anti-EGFR and anti-poly-ubiquitin. Representative of results from 3 biological replicates. (**L** to **N**) HSC-5 cells were transfected with plasmid expressing ADRM1. Total cell lysates were subjected to immunoblotting with anti-CCDC40, anti-ADRM1, anti-UCH37, anti-KTN1, anti-EGFR and anti-poly-ubiquitin. Representative of results from 3 biological replicates.


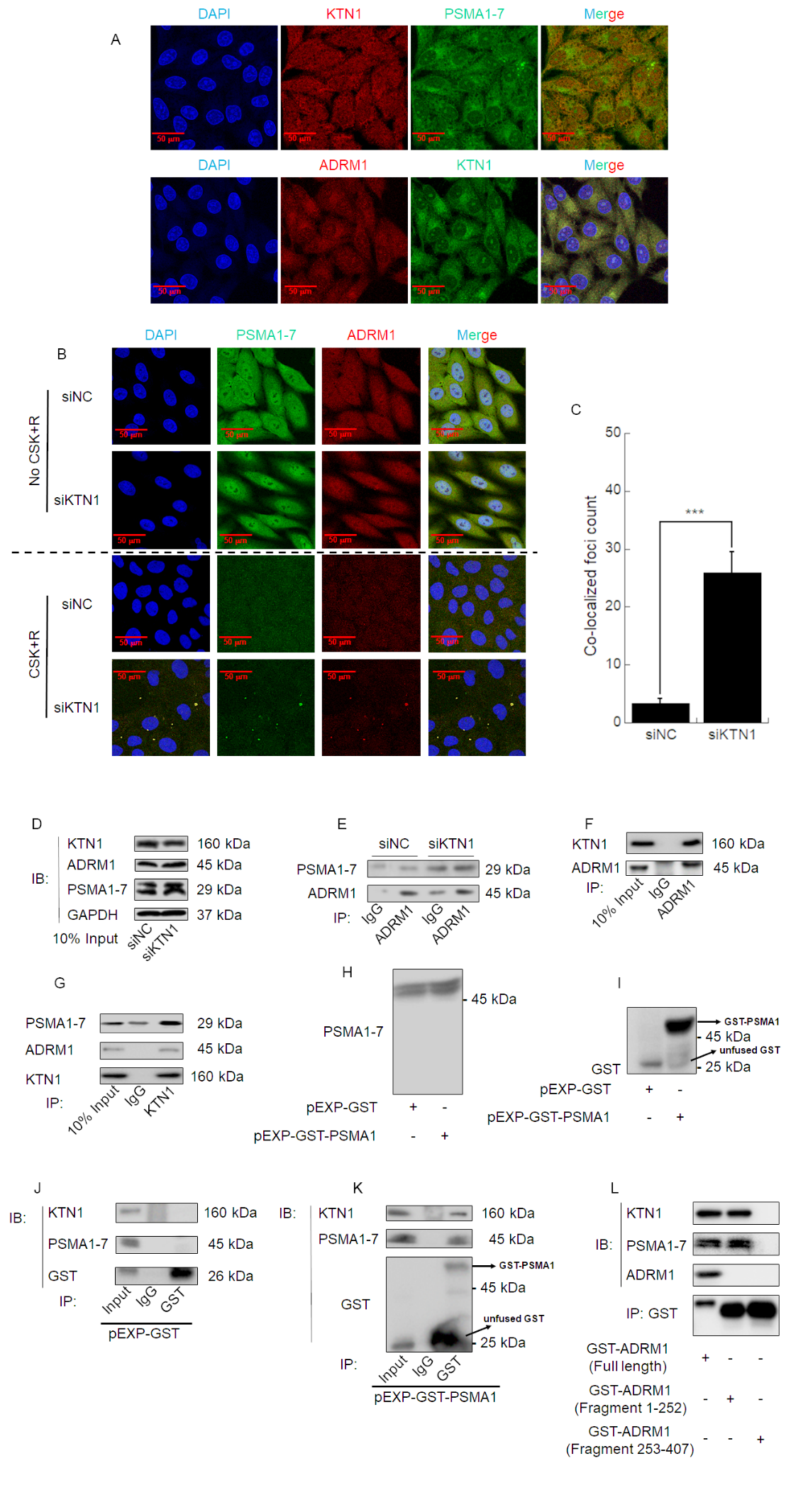


**Fig. S5. KTN1 and PSMA1 interact with each other and competitively bind ADRM1 at amino acid residues Met1–Ala252 in HSC-5 cells.**

(**A**) Confocal immunofluorescent staining images showing co-localization between KTN1 and PSMA1-7 or ADRM1 in HSC-5 cells. Representative of results from 3 biological replicates. (**B and C**) HSC-5 cells were transfected with siKTN1 or siNC and then were treated with or without CSK buffer. Confocal immunofluorescent staining images show co-localization between PSMA1-7 and ADRM1 in A431 cells. Quantified foci count represents the means ± SD. Representative of results from 3 biological replicates. (**D** and **E**) HSC-5 cells were transfected with siKTN1 or siNC. Total cell lysates were subjected to Co-IP and immunoblotting with anti-PSMA1-7, anti-ADRM1 and anti-KTN1. Representative of results from 3 biological replicates. (**F** and **G**) HSC-5 cell lysates were subjected to Co-IP analysis. Representative of results from 3 biological replicates. (**H** to **K**) HSC-5 cells were transfected with plasmid expressing GST or GST-tagged PSMA1(GST-PSMA1). Total cell lysates were subjected to Co-IP and immunoblotting with anti-GST, anti-PSMA1-7 and anti-KTN1. Representative of results from 3 biological replicates. (**L**) HSC-5 cells were transfected with plasmid expressing GST-tagged full length ADRM1, GST-tagged ADRM1 1-252 amino acid residues or GST-tagged ADRM1 253-407 amino acid residues. Total cell lysates were subjected to Co-IP and immunoblotting with anti-GST, anti-ADRM1, anti-PSMA1-7 and anti-KTN1. Representative of results from 3 biological replicates.


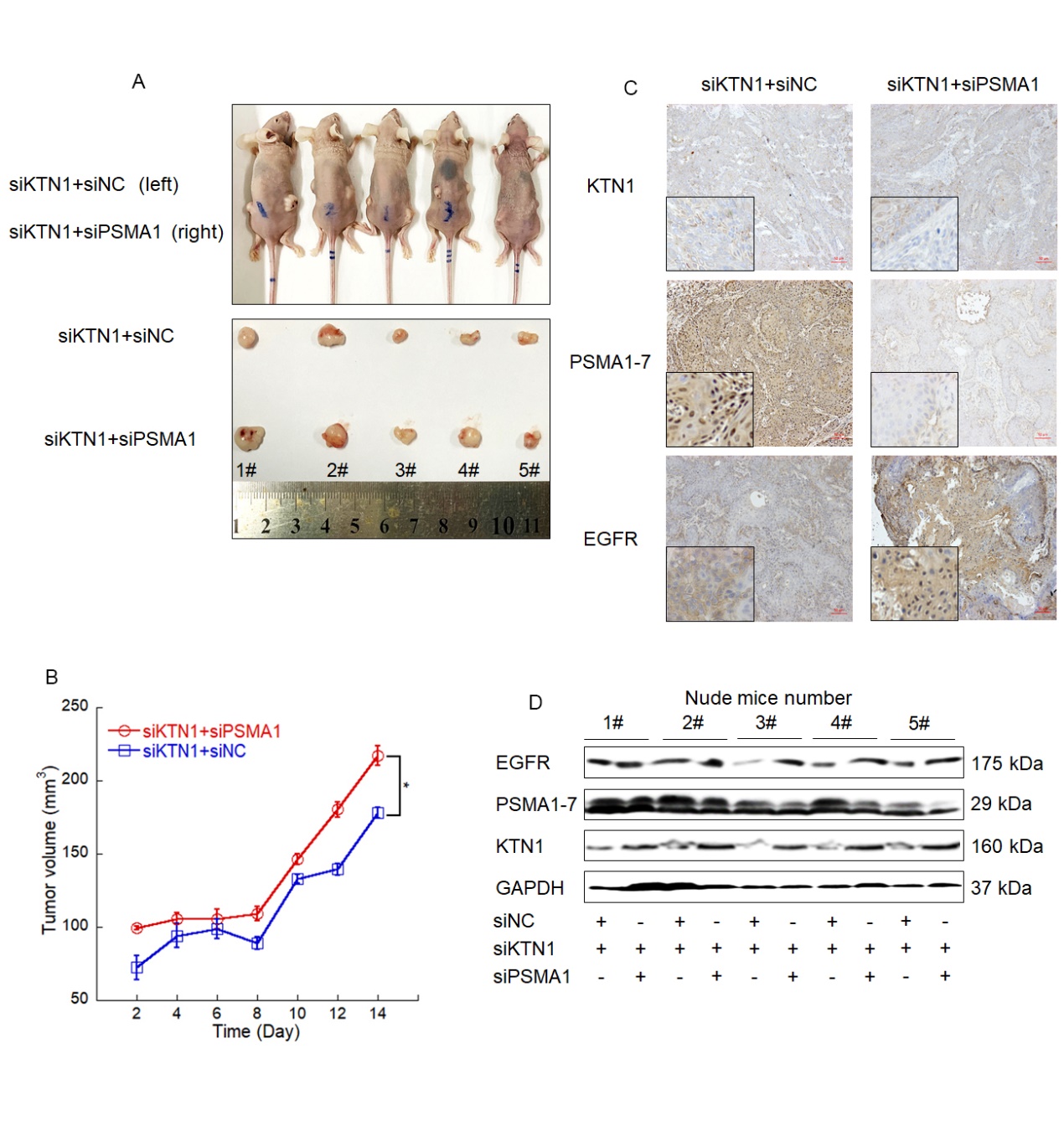


**Fig. S6. PSMA1 represents a key modulator in KTN1-mediated EGFR degradation in *vivo*.**

(**A** and **B**) Tumorigenicity of PSMA1 depletion in siKTN1-transfected A431 cells in nude mice. siKTN1 transfected or siPSMA1 and siPSMA1 co-transfected A431 cells (0.1mL; 2×10^6^ cells) were injected into different side of nude mice. Tumor growth was measured every 2 days. 2 weeks later, the mice were sacrificed to collect the tumor samples. Photo credit: Ji Ma, Department of Radiation medicine, School of Public Health, Southern Medical University. Tumor volumes represent the means ± SD, n = 7 for each group. (**C**) Representative photos of immunohistochemistry (IHC) staining of xenografted tumors with anti-PSMA1-7, anti-EGFR or anti-KTN1. (**D**) Lysates of the xenografted tumors were subjected to immunoblotting with anti-PSMA1-7, anti-EGFR and anti-KTN1.


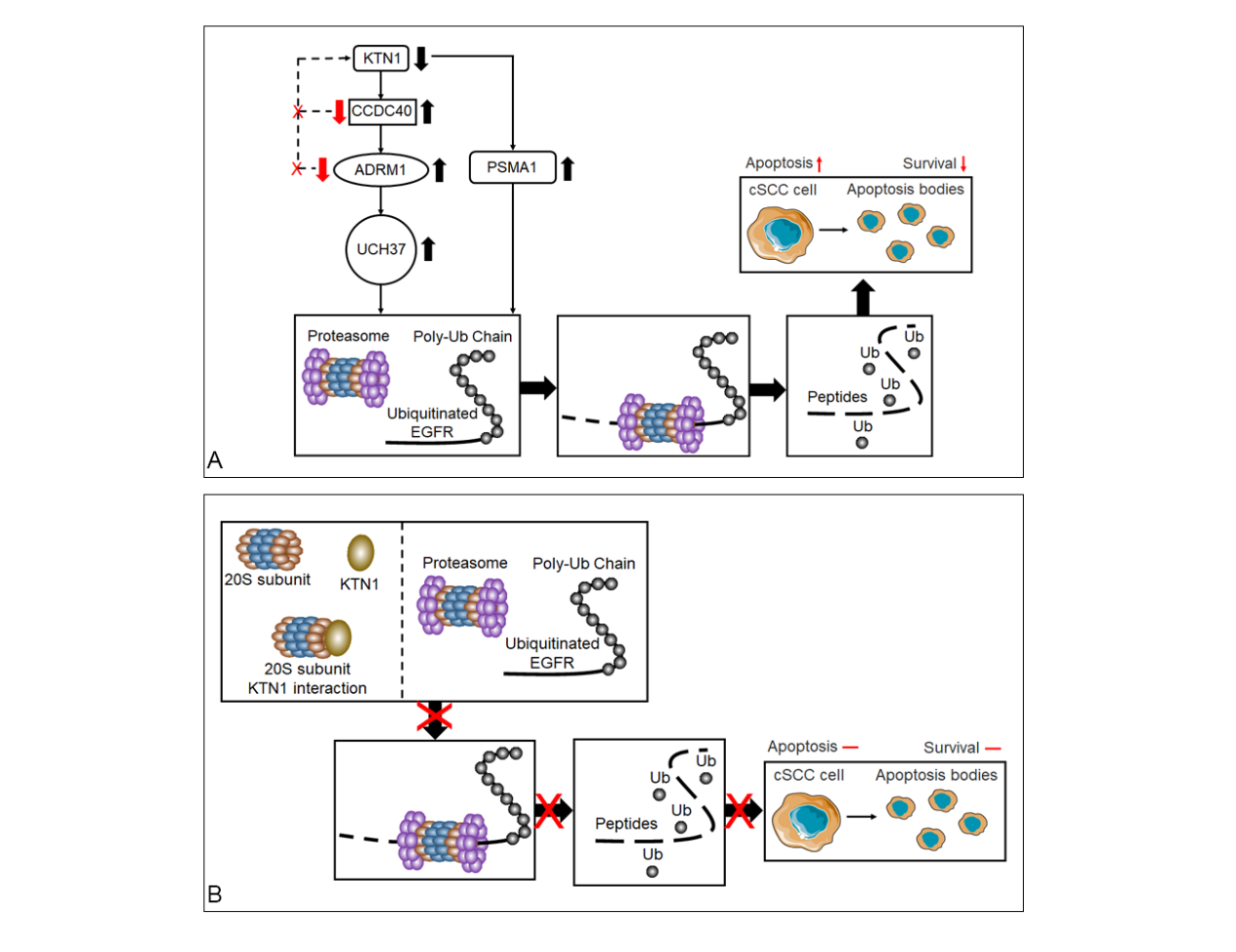


**Fig.S7. Graphical abstract of this study.**

(**A**) Knockdown of *KTN1* induces the EGFR degradation in cSCC cells by increasing the expression of PSMA1 and triggering the CCDC40/ADRM1/UCH37 regulatory axis to activate the UPS. (**B**) KTN1 competitively interacts with the 20S CP to curb proteasome 26/30S formation, resulting in normal levels of EGFR protein.

**Table S1.** Results from iTRAQ analysis of differentially expressed proteins.

**Table S2.** Sequences of siRNAs, primers and EMSA biotin-labelled probes.

| Table S2. Sequences of siRNAs, primers and EMSA biotin-labelled probes | | | |
| --- | --- | --- | --- |
| **Primers** |  | Forward (5' → 3') | Reverse (5' → 3') |
| 20S α | PSMA1 | GCCTGTGTCTCGTCTTGTATCTCTAA | TCCGGCCATATCGTTGTGTT |
|  | PSMA2 | TGAATATGCTTTGGCTGCTGTAG | CCACACCATTTGCAGCTTTAATT |
|  | PSMA3 | GAATGACGGTGCGCAACTCT | CAGCCCCAATAACCGTATGAA |
|  | PSMA4 | ACTATATTTTCTCCAGAAGGTCGCTTA | CAAACAGGTGCCTGCATGTC |
|  | PSMA5 | CCAGAGTGGAGACACAGAACCA | GGGTCACACTCTCCACTGTCATT |
|  | PSMA6 | CCCGAGGGTCGGCTCTA | GATGTAAGGCCACCCTGGTTAA |
|  | PSMA7 | GCCAGTCTGAAGCAGCGTTAT | TGAGGGCAGAGATGCCAAAC |
| 20S-β | PSMB1 | AGGCGCTTCTTTCCATACTATGTT | GCCCCCTTTCCTTCTTCATC |
|  | PSMB2 | GATGCGAAATGGATATGAATTGTC | AGGTTTCGGCGTGTGAAGTT |
|  | PSMB3 | CTGTGGACCGGGATGCA | GATTTTGTCCTTCTCGATGATGTG |
|  | PSMB4 | ACATGCTTGGTGTAGCCTATGAAG | AGAGGCTGAGCCAAGTATGCA |
|  | PSMB5 | CAGAAGAGCCAGGAATCGAAA | TCCATGGCGGAACTTGAAG |
|  | PSMB6 | CAAGGAAGAGTGTCTGCAATTCA | CCGCTCCATGGCCAAA |
|  | PSMB7 | CAGCCAATCGGATGCTGAA | AGGGCTGCACCAATGTAACC |
| 19S ATPases | PSMC1 | AGACCAGGCCGCATTGAC | TGCGCTTCTTCGTCTTTTCA |
|  | PSMC2 | TTGGCTGCAGATAAGCAGACA | TCTTTGTACACCTGGCAACCTGTA |
|  | PSMC3 | CACACGGCTGCTGGACAGT | CTTGGAGCTCATGGGTGACTCT |
|  | PSMC4 | GGCCCGGCCAGATAAGATT | CAACATTCCACTCTCCTGACAGAT |
|  | PSMC5 | CGAGAACGGCGAGTCCAT | TTCTGCATGACCTTGGCTACTG |
|  | PSMC6 | CCAGAGTTATTTCAGCGTGTAGGA | CGTACCTGGTGGTCCATATAACAA |
| 19S non-ATPases | PSMD1 | GCTTCTGTGCCTGGATCCA | TCCATCGAGTCACTGTCTTTCTCT |
|  | PSMD2 | GCGTGAGTGCCTCAAGTATCG | CCAGATGCCTGACATACTCATGA |
|  | PSMD3 | GAGCAGGCCAACAACAATGA | GCTTTGATTCGCCCTGTGTAG |
|  | PSMD4 | CACTATGGTGTGTGTGGACAACAG | TGCAGCCTGGTGGGTAAGA |
|  | PSMD6 | TGACAAAGAGGGAGCTCTGACA | GACCCAGGGCCACAGTTTT |
|  | PSMD7 | GAAGCTGAGGAAGTTGGAGTTGA | TGCCCACCGTCGTGTCTT |
|  | PSMD11 | GCCATCTACTGCCCCCCTAA | ATGGATAATACCCGACTGCATGT |
|  | PSMD12 | GACTCGTACTGCTTCCGATATGG | TCATAGCACATCTTCACTACTGCAACT |
|  | ADRM1 | GGGTCCAAGCGGCTTTTC | GCTCCTCATCCTGGTCTGTCTT |
|  | PSMD14 | TGAACAGCTGGCAATAAAGAATG | AGTACATCCACATGTTCCTCCAAA |
|  | GAPDH | GGATATTGTTGCCATCAATGACC | AGCCTTCTCCATGGTGGTGAAGA |
|  | KTN1 | ATGCAGTTGAACACCAGAGGAAGA | ATGCAACCATTCACCATAACTCAAA |
|  | EGFR | CGAGGGCAAATACAGCTT | AAATTCACCAATACCTATT |
|  | CCDC40 | GTCGCCTAGCAACGGGAAAT | CTCTTCCCCTTCCACTGCTG |
|  | GAPDH | GGATATTGTTGCCATCAATGACC | AGCCTTCTCCATGGTGGTGAAGA |
| **siRNAs** |  |  |  |
|  | siKTN1 | GATGGATAATGCTGACTCA |  |
|  | siKTN1# | GAGTGATCTTTCTAGCAAA |  |
|  | siEGFR-1 | GAGGAAATATGTACTACGA |  |
|  | siEGFR-2 | GGAGCGAATTCCTTTGGAA |  |
|  | siADRM1 | GCCGGAAAGTCAACGAGTA |  |
|  | siCCDC40 | CCTGGAGCTTGAAATCAAA |  |
|  | siCCDC40# | CGACCCTCCTGAATCAACT |  |
|  | siPSMA1 | TCCAGATTTGTATTCGATA |  |
|  | siPSMA1# | TTTATGCGTCAGGAGTGTT |  |
|  | siUCH37 | GAAGGTGAAATTCGATTTA |  |
|  | siUCH37# | GCCAGTTCATGGGTTAATT |  |
|  | siNC, 5 nmol (siN0000001-1-5, Guangzhou RiboBio Co., Ltd) | | |
| **Biotin labled probes for EMSA** |  |  |  |
| 5' biotin | Native probe | ggaaaagcgctcgccatgcgcggggtccccggcccccggt | |
| 5' biotin | Mutant probe | ggaaaagcgctcgccatgaacggggtccccggcccccggt | |
